# A Facile Glycerol-Assisted Synthesis of Low-Cu^2+^-Doped CoFe_2_O_4_ for Electrochemical Sensing of Acetaminophen

**DOI:** 10.3390/bios13120997

**Published:** 2023-11-23

**Authors:** José Guillermo Alfonso-González, Claudia Patricia Granja-Banguera, Jimmy Alexander Morales-Morales, Andrés Dector

**Affiliations:** 1 Grupo de Investigación en Química y Biotecnología (QUIBIO), Facultad de Ciencias Básicas, Campus Pampalinda, Universidad Santiago de Cali, Cali 760035, Colombia; jose.alfonso00@usc.edu.co (J.G.A.-G.); claudia.granja00@usc.edu.co (C.P.G.-B.); 2 CONAHCYT (Consejo Nacional de Humanidades, Ciencias y Tecnologías), Universidad Tecnológica de San Juan del Río, San Juan del Río 76800, Querétaro, Mexico; adector@conahcyt.mx

**Keywords:** urine, modifier, sensor, spinel, electrooxidation

## Abstract

This work devised a simple glycerol-assisted synthesis of a low-Cu^2+^-doped CoFe_2_O_4_ and the electrochemical detection of acetaminophen (AC). During the synthesis, several polyalcohols were tested, indicating the efficiency of glycerin as a cosolvent, aiding in the creation of electrode-modifier nanomaterials. A duration of standing time (eight hours) before calcination produces a decrease in the secondary phase of hematite. The synthesized material was used as an electrode material in the detection of AC. In acidic conditions (pH 2.5), the limit of detection (LOD) was 99.4 nM, while the limit of quantification (LOQ) was found to be (331 nM). The relative standard deviation (RSD), 3.31%, was computed. The enhanced electrocatalytic activity of a low-Cu^2+^-doped CoFe_2_O_4_-modified electrode Cu_0.13_Co_0.87_Fe_2_O_4_/GCE corresponds extremely well with its resistance R_ct_, which was determined using the electrochemical impedance spectroscopy (EIS) technique and defined its electron transfer capacity. The possibility of a low-Cu^2+^-doped CoFe_2_O_4_ for the electrochemical sensing of AC in human urine samples was studied. The recovery rates ranging from 96.5 to 101.0% were obtained. These findings suggested that the Cu_0.13_Co_0.87_Fe_2_O_4_/GCE sensor has outstanding practicability and could be utilized to detect AC content in real complex biological samples.

## 1. Introduction

Acetaminophen (AC) is a drug with wide application as an analgesic medicine for the treatment of a fever. It is also the alternative to aspirin, particularly for sick people who experience adverse reactions to aspirin [1]. Additionally, the AC is classified as an emerging pollutant present in water sources [2].

In recent years, different methods have been identified for the detection or quantification of AC: highly sophisticated chromatographic used for the simultaneous detection and quantification of AC with other substances [3,4], capillary electrophoresis-mass spectrometry [5], and vibrational methods in commercial AC tablets, where the unknown nature of the excipients used negatively influenced the accuracy of the method [6,7,8]. Also, the spectrofluorimetric method at ultra-trace levels where an aqueous mixture of sulfuric acid was used. The method was inappropriate due to this reagent which was a controlled narcotic [9,10]. Finally, radiometric fluorescent sensors for the rapid detection of AC where hydrochloric acid was used during the experimental methodology were also used [11]. For this reason, electrochemical detection is a competitive and attractive alternative methodology, due to the attributes of simple instrumentation, high sensitivity, low cost, and green chemistry methods.

For the investigation of electroactive substances in physiological fluids and pharmaceutical samples, electrochemical methods have been widely used. Among the electrochemical methods is cyclic voltammetry (CV), which offers great versatility to study electroactive substances. Currently, a wide variety of nanomaterials have been designed for the modification of electroactive surfaces, and to optimize the determination of electroactive compounds of interest by increasing their surface and adsorption sites, improving the selective interactions between the electrode and the substance, and achieving a greater sensitivity and performance [12,13,14,15,16,17].

A diversity of nanomaterials based on ferrite-type mixed metal oxides have been synthesized by the scientific community, interested in exploring their properties and improving them. In this sense, the ferrite compounds are active materials to improve the performance of the detection of electroactive substances.

Several procedures have been developed to obtain ferrite nanoparticles [18,19,20,21]. The influence of cosolvents or chelating agents and preparation times have been studied, giving interesting results, where it has been sought to minimize the agglomeration of particles and control their size and morphologies [22,23,24,25,26,27,28,29,30,31,32]. In this case, the citrate sol-gel combustion method has been a simple methodology used for a long time.

Interlinking copper-cobalt ferrite nanoparticles, which have exceptional physicochemical properties making it a promising material for applications such as the sensing of relevant chemical species, have been reported [33,34]. The synthesis and characteristics of mixed cobalt-copper ferrites have been the subject of several papers during the last three years. As an illustration, Khairy et al. [35] used the self-combustion process with urea as fuel to synthesize both pure and mixed ternary ferrite nanocrystallites Cu_0.5_Co_0.5_Fe_2_O_4_ (CuCoF). They concluded that the magnetic, structural, and electrochemical characteristics of the ferrites that were produced were significantly influenced by the distribution of cations in the examined samples. Dadaei et al. [36] illustrated the benefits, including the simple and effective method of separating non-toxic products and the minimal amounts of catalyst that were needed because they were easily recyclable and reusable, which made it possible to synthesize organic chemicals in an economical and environmentally friendly way. Ding et al. [37] used a one-step hydrothermal procedure to successfully manufacture Co_x_Cu_1−x_Fe_2_O_4_/RGO composites. They observed that adjusting the doping rate with Co^2+^ and Cu^2+^ ions might improve the composite material’s microwave radiation adsorption capabilities, making it a suitable technique for the creation of microwave-absorbing materials. Aswad et al. [38] used laser-assisted hydrothermal synthesis (LAHS) methods to create cobalt-copper ferrite nanoparticles Co_1−x_Cu_x_Fe_2_O_4_ (x = 0, 0.3, and 0.7), and the results demonstrated that the cation distribution and particle size were directly related to the magnetic properties. Furthermore, the antibacterial ability of the produced nanoparticles against S. aureus and E. coli was improved after the substitution of Cu into cobalt ferrite. Patil et al. [39] used a solid-state reaction to create a series of Cu-Co ferrite-based bulk particles (Cu_(1−x)_Co_(x)_Fe_2_O_4_) with the x varying from 0.0 to 0.6. Using Raman spectroscopy, they evaluated the effect of increasing the Co concentration on the cubic copper ferrite (CuFe_2_O_4_) structure. They discovered that when Co^2+^ ions were added to the CuFe_2_O_4_ structure, the lattice parameter dropped when compared to the original structure. The Raman scattering spectra revealed that the Co, Cu, and Fe ions occupy the octahedral sites, while the Fe ions occupy the tetrahedral sites. Mahmood et al. [40], used the free software Visualization Electronic and Structural Analysis (VESTA 3) and Mercury 4.0, a free program created by the Cambridge Crystallographic Data Center, to design crystallography information files (CIF) formed by solid solution of Co_x_Cu_1-x_Fe_2_O_4_ with the substitution factor x = 0 to 1 with an increment of 0.1 depending on Vegard’s Law. They looked at several parameter correlations, including how the replacement factor affected the network parameter, the unit cell’s volume, and its density. The XRD revealed a small shift in the peak position because of the lattice size change, and a small shift in the peak intensity because of the Cu concentration change, which increased the electron density. Anju et al. [41] used a sonochemical approach to create spinel ferrite nanoparticles Cu_0.33_Co_0.67_Fe_2_O_4_ (CuCoF1) and Cu_0.67_Co_0.33_Fe_2_O_4_ (CuCoF2). The ferrites produced exhibited conventional ferromagnetic properties. Venkateshwarlu et al. [42] employed a two-fold sintering ceramic process to create copper ferrites doped with the Cu_1−x_Co_x_Fe_2_O_4_ compositions (x = 0.2, 0.4, and 0.6). They looked at its electrical conductivity characteristics at different temperatures and compositions. It was established by them that when the cobalt content in doped copper ferrite increases the electrical conductivity values drop. Patil et al. [43] successfully created Co_1−x_Cu_x_Fe_2_O_4_ spinel ferrite samples using a solid-state synthesis method, where x = 0.00, 0.25, 0.5, 0.75, and 1.00. Given their superior resistive nature, the ferrites’ AC conductivity studies suggested that these samples could be dielectric materials. Al Kiey et al. [44] used citrate as a precursor to create the Co/CuFe_2_O_4_ transition metal mixed ferrite nanoparticles. They observed that the Co/CuFe_2_O_4_ ferrite has the highest specific capacitance when compared to other metal ferrite nanoparticles. Alzoubi et al. [45] found that Co^2+^-doped copper ferrite nanoparticles, or Cu_0.75_Co_0.25_Fe_2_O_4_, were created via a hydrothermal process. CuFe_2_O_4_, a pure copper ferrite, was studied to see how Co^2+^ affected its structure and magnetic characteristics. In comparison to the pure Cu ferrite, they found that adding the Co to the copper ferrite lowers the density and raises the lattice constant. Hunyek et al. [46] found that for the sol-gel synthesis of the cobalt-copper ferrite nanoparticles, an aqueous solution of tapioca starch was used as a chelating agent. This material exhibited the lowest coercive field and the highest saturation magnetization. Mohamed et al. [47] recently reported the impact of the Co^2+^ exchange for Cu^2+^ on the optical, structural, and magnetic characteristics of Co_1−x_Cu_x_Fe_2_O_4_ nanoparticles. The exchange of the Cu^2+^ ions for Co^2+^ allowed for variations in properties such as optical, magnetic, the size of its nanocrystals, and the energy gap. These variations in properties have a great benefit in the use of these compounds for many potential applications. Saleem et al. [48] prepared Cu^2+^-doped CoFe_2_O_4_ nanoparticles (CFO NPs) using sol-gel synthesis. Their studies demonstrated that the electrical properties of nanomaterials could be improved by doping them with a small number of Cu^2+^ ions. The contamination of Co ferrites with a small concentration of Cu^2+^ ions improved the structural and electrical characteristics and made it a material with a potential application for the development of diode technology, sensors, and electrical devices.

The previous investigators did not report in their synthesis methodology that they used propylene glycol or glycerin as a cosolvent. Additionally, it was evidenced that there are no reports of these ferrites as material for electrochemical detection. Our group has recently reported the synthesis of copper and cobalt ferrites where previous tests were performed using propylene glycol [49]. Now our group has reviewed the assisted synthesis with a glycerin (G) cosolvent, comparing the results obtained and expanding its application to biological matrices, such as urine.

This article describes a facile citrate sol-gel combustion method to prepare low-Cu^2+^-doped CoFe_2_O_4_ ferrite nanoparticles (LCF NPs). The results of the effect of the hydroxylated compound, minimal quantity of copper, and standing time in obtaining the LCF NPs are presented. The material obtained was used in the electrochemical detection of AC. This is the first report on the development of low-Cu^2+^-doped CoFe_2_O_4_ ferrite nanoparticles with potential applications in the quantification of drugs in complex biological media.

## 2. Materials and Methods

### 2.1. Structural Properties of Low-Cu^2+^-Doped CoFe_2_O_4_ Ferrite Nanoparticles, LCF NPs

Cobaltous chloride hexahydrate CoCl_2_·6H_2_O, ferric chloride anhydrous, FeCl_3_, Cupric chloride dihydrate CuCl_2_·2H_2_O, citric acid, C_6_H_8_O_7_, propylene glycol (C_3_H_8_O_2_) and glycerin (C_3_H_8_O_3_), and ammonium hydroxide NH_4_OH were used as the starting materials and were of a reagent grade. The low-Cu^2+^-doped CoFe_2_O_4_ ferrite (Cu_x_Co_1−x_Fe_2_O_4_) with a composition of x = 0.09 (S-009), 0.13 (S-013), and 0.28 (S-028) was synthesized using acid combustion, the sol-gel citrate method [49].

To synthesize LCF NPs, two 30 mL solutions, A and B, were produced separately in deionized water with a conductivity of 0.05 µs cm^−1^ to obtain homogeneous solutions. Solution A was made up of stoichiometric quantities of cupric chloride dihydrate (CuCl_2_·2H_2_O) and cobalt chloride hexahydrate (CoCl_2_·6H_2_O) diluted in 30 mL of deionized water. Solution B was made in the same way, with stoichiometric amounts of ferric chloride anhydrous, FeCl_3_, and a 1:1 relationship with the molar amount of citric acid C_6_H_8_O_7_, both dissolved in the same amount of deionized water. Solution A was added dropwise to solution B, and during the mixing of those solutions, it was stirred continuously for 20 min, and for an additional half hour. Ammonia hydroxide was added drop by drop to the mixture to adjust the pH value to 7 as exhibited in Figure S1.

Subsequently, it was heated for half an hour at 50 °C, and 10 mL of propylene glycol (C_3_H_8_O_2_) or glycerin (C_3_H_8_O_3_) at 10% by volume was added dropwise to the solution. It was evaporated from 80 to 120 °C and then placed in the muffle for two hours at 450 °C, then macerated for 10 min and calcined for three hours at 950 °C. Thus, the spinel-type low-Cu^2+^-doped CoFe_2_O_4_ LCF NPs S-028 and S-009 samples were obtained with 10% propylene glycol (P) and 10% glycerin (G), respectively.

Additionally, the synthesis procedure was developed, with 10% glycerin but incorporating a standing time of 8 h (S-013 sample) between heating at 120 °C and calcination at 450 °C. Finally, the powders were washed with diluted 10 mM HCl, and sufficient deionized water, and dried at 100 °C in an oven.

X-ray diffraction (XRD) patterns were obtained using a Malvern Panalytical Empyrean device in a beam-embedded configuration with a detector scintillator and a parallel plate collimator to investigate the structural parameters, phase identification, and chemical purity of the LCF NPs samples. The surface morphology micrographs and particle size distribution of investigated nano-spinel ferrites were revealed by FEI Quanta 650 FEG field emission scanning electron microscope produced by FEI (USA), equipped with EDX (Apollo XL, EDAX) and a Transmission electron Microscope (TEM) FEI Tecnai F20 Super Twin (FEI, Hillsboro, OR, USA) analyzer, respectively. The thermal gravimetric analysis and differential scanning calorimetry (TGA-DSC) were performed with a Discovery SDT650 TA instrument (New Castle, DE, USA) at a temperature ramp of 283 K/min, up to 1300 °C. The phase identification and chemical purity of investigated samples were reassessed using an Attenuated total reflectance with Fourier transform infrared spectroscopy (ATR-FTIR, JASCO FT/IR-4100, Japan, Tokyo), with a resolution of 4 cm^−1^ and ATR source (PRO450-S), a scan of 650–4100 cm^−1^ with 512 scans, and a Raman spectrophotometer (DXR Smart Raman device, Thermo Fisher Scientific, Waltham, MA, USA), with a range of 50 to 3370 cm^−1^, containing a refrigerated CCD detector (−51 °C), with a diode laser of (785 nm).

### 2.2. Electrochemical Measurements

A Bioanalytical Systems, Inc., BAS Inc. (West Lafayette, IN, USA)single-compartment cell with three electrode arrangements was employed. The reference electrode was an Ag/AgCl electrode (MF-2056, BAS Inc.) and the auxiliary electrode was a 23 cm long coiled platinum wire (0.5 mm diameter) electrode (MW-1033, BAS Inc.). In this study, all potential values were reported relative to an Ag/AgCl electrode. Working electrodes are a glassy carbon electrode (GCE) (MF-2012, BAS Inc.), 3 mm in diameter, and an LCF NPs modified GCE electrode. Reference material N-acetyl-p-aminophenol (AC) (Merck, GA grade, Darmstadt, Germany). The buffer solution (supporting electrolyte) was made up of 0.1 M KH_2_PO_4_ (Merck GA grade), in deionized water. A stock AC solution was prepared before each electrochemical measurement.

To optimize the electrode surface modification process, tests were done with different quantities and concentrations of LCN NPs on the electroactive surface of a GCE electrode (MF-2012, BAS Inc.). Modified and bare electrodes were also studied to determine the electron transfer rate using a 2.5 mM solution of [Fe(CN)_6_]^3−/4−^ in 0.1 M KCl. The electrode surfaces were pretreated by cleaning processes and dried at 25 °C for 1 h in an argon atmosphere [48]. All electrodes were kept in a vacuum desiccator.

To detect the AC content in real human urine samples, the standard addition method was used. Sample pretreatment consisted of filtering through PTFE polytetrafluoroethylene membranes. First, they were filtered with a 0.45 µm PTFE membrane and then with a 0.2 µm PTFE membrane. The calibration curve approach was used to test the possibility of measuring AC in human urine samples. To demonstrate that matrix components do not interfere with AC detection, model human urine samples were employed. As a result, when evaluating human urine samples, we did not need to employ a solid phase extraction, which is generally used for the preliminary separation. The urine sample was diluted up to 100 times with PBS medium (pH = 2.5) and used for analysis without any prior treatment. Separately, 5 mL of this urine sample solution was taken and enriched with 2.5 mL of acetaminophen solutions with known concentrations (8.0, 16.0, 32.0, 48.0, and 64.0 µM) diluted to a volume of 10 mL with medium buffered PBS (pH = 2.5) and used for a differential pulse voltammetry (DPV) measurement.

For electrochemical analyses, an Autolab PGSTAT 128N potentiostat/galvanostat (Metrohm, Herisau, Switzerland) was used. Electrochemical studies were mainly developed using conventional electrochemical techniques. In this regard, cyclic voltammetry CV was utilized to evaluate the electrochemical behavior of AC in the electrodes modified with various LCF NPs and in a potential window ranging from 0.1 V to 1.3 V. In addition, the influence of the scan rate was investigated. in the range of 25 to 350 mVs^−1^, and a differential pulse voltammetry DPV under predetermined parameters (a pulse time of 50 ms, a step potential of 5 mV, a scan rate of 10 mVs^−1^, and pulse amplitudes of 100 mV) was employed for the analyzes of AC in human urine samples [48]. The electrochemical impedance spectroscopy (EIS) technique, where frequencies from 0.1 Hz to 100 kHz were necessary, was employed to study the electron transport of the modified and unmodified electrodes with LCF NPs. A temperature of 29.0 °C was the working condition during all experiments unless otherwise specified.

## 3. Results and Discussion

### 3.1. Synthesis of Low-Cu^2+^-Doped CoFe_2_O_4_ Nanoparticles LCF NPs

#### 3.1.1. Structural Properties

**Figure 1:**
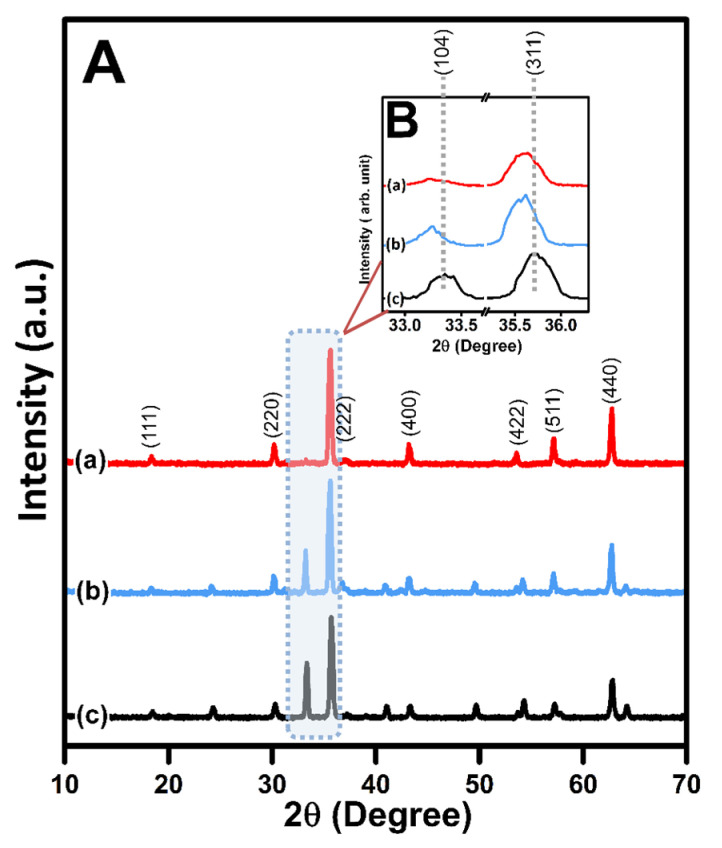
(**A**) XRD patterns of LCF NPs. Effect of hydroxylated agents (G or P) and standing time (t_s_). (a) + G and t_s_ = 8-h, S-013; (b) + G and t_s_ = 0, S-009 and (c) + P and t_s_ = 0, S-028, (**B**) Insert produced using XRD patterns.

Figure 1, shows the XRD patterns for the spinel-type ferrites substituted with copper and cobalt, using a different cosolvent.

Figure 1A(c) shows the XRD pattern results for S-028 sample obtained with 10% propylene glycol (P). A good number of two-phase signals were found: eight signals in the XRD patterns at 18.45° (111), 30.35° (220), 35.68° (311), 37.13° (222), 43.22° (400), 53.60° (422), 57.20° (511), and 62.87° (440) confirming the formation mainly of spinel-type ferrite phases (JCPDS 00-034-0425). These XRD peaks signify the cubic spinel of the space group Fd^3^m [49]. A hematite-type secondary phase (JCPDS:01-089-2810) was evidenced, with six secondary phase signals (all marked with an asterisk) [50,51,52]. Changing the propylene glycol (P) to glycerin (G) showed different XRD patterns in Figure 1A(c) and 1A(b), respectively. In Figure 1A(b) the hematite signal was affected by the cosolvent change, decreasing its intensity. The XRD pattern in Figure 1A(a) showed that a longer standing time (eight hours) before calcination eliminated the secondary phase of hematite. This result demonstrated the advantage of using glycerin (G) and standing time to make spinel-type ferrite more efficient. In Figure 1B, the insert showed two signals, (311) for spinel-type ferrite phases and one marked with an asterisk (104) for hematite, respectively. Changes in the width of the peaks and shifts for each peak were observed. The displacement of the peaks to higher degree values (0.16° and 0.10°) is the result of the incorporation of copper species in the cobalt ferrite phase and hematite phase.

**Figure 2:**
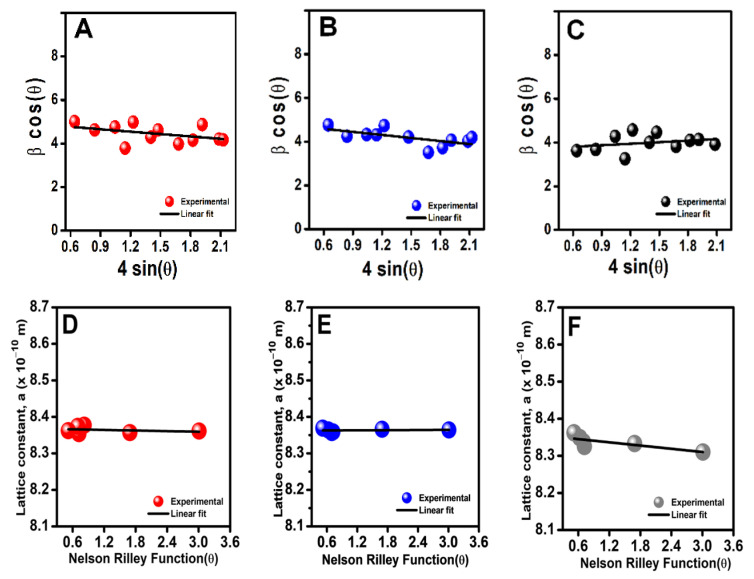
The W–H plots of the investigated LCF NPs. (**A**) S-013, (**B**) S-009, and (**C**) S-028 samples. Β cos(θ) × 10^−3^. The calculated values of lattice constant a vs. the Nelson Riley function F(θ) for the investigated nanoparticles. (**D**) S-013, (**E**) S-009, and (**F**) S-028 samples.

From the obtained XRD spectra, the amplitude of the X-ray diffraction peaks was used, with the help of the Williamson-Hall (W-H), Bragg’s, and Nelson Riley approaches, to obtain microstructural parameters such as microstrain (ε), crystallite size (D), and lattice constant (a), for LCF NPs previously synthesized. The Williamson–Hall model (W-H) is a simplified integral breadth method that separates the contributions from diffraction line broadening. The crystallite sizes (L) and lattice microstrain (ε) were determined from XRD using the Williamson–Hall method [45,46,47,48,49,50,51,52].

(1) $$\frac{\beta \cos\theta }{\lambda }=\frac{k}{L}+\frac{4\varepsilon \sin\theta }{\lambda }$$

where, λ, the X-ray wavelength (0.15418 nm), ε, is the mean value of internal strain values related to the nanoparticles; k is the shape factor value (0.94) and θ, is the diffraction angle; β, the full width at half maximum (FWHM) of diffraction peak [52]. The Williamson-Hall plot for LCF NPs nanoparticles is shown in Figure 2.

In Figure 2A–C, a progressive increase in the intersection values is observed and the values of the size of the crystallites are measured from the intersection of the lines using Figure 2. Furthermore, using the same Figure, it was possible to know the size of the nanoparticles 29.0 nm for S-013, 29.7 nm for S-009, and 39.5 nm for S-028. The slope of the curve allowed the average microstrain values to be measured from Figure 2. The increase in particle size is also followed by an increase in microstrain (ε) from −4.7 × 10^−4^ at S-009 to 2.3 × 10^−4^ at S-028 samples as indicated in Table 1. These results demonstrated a relationship between lattice defects and the proportion of Cu^2+^ ions in the crystal lattice of LCF NPs. The defects increase as the proportion of Cu^2+^ ions increase. The negative slope of Figure 2A,B indicates that the existing stress due to the radii mismatch of Cu^2+^ and Co^2+^ ions and cationic vacancies is responsible for the compressive microstrain in the samples [53]. From Figure 2, it was shown that the slopes of Figure 2A of the pure S-013 and Figure 2B of S-009 nanoparticles, were negative, indicating the presence of deformation due to compression in those nanoparticles of smaller size. In the case of nanoparticles S-028 the slope was positive indicating deformation due to traction [52,54]. Therefore, as doping of a greater amount of Cu^2+^ ions occur, the slope values change sign, indicating that the crystal lattice is expanding, changing the compressive strain to tensile strain [52,54].

The lattice constant (a) was determined from the XRD, Bragg’s, and Nelson Riley approaches as follows:

(2) $$a=\frac{\lambda }{2}\sqrt{\frac{h^{2}+k^{2}+l^{2}}{{sin}^{2}\theta }}$$

where (a) is the lattice constant, λ is the wavelength, (h k l) is Miller indices and θ is the position of the peak, respectively [55,56]. The (a) value for each sample extracted from the prominent XRD line (hkl = 311) with the maximum peak intensity [46]. The (a) values were plotted against Nelson Riley function F(θ)

(3) $$F\left(\theta \right)=\frac{{cos}^{2}\theta }{2}\left(\frac{1}{\theta }+\frac{1}{sin\theta }\right)$$

The precise lattice parameter of LCF NPs was calculated with the support of linear regression analysis between the lattice parameter and the Nelson-Riley function. Figure 2D–F shows the typical Nelson-Riley plots and parameters for LCF NPs. The value of the lattice constant (a) calculated from Equation (2) for S-013, S-009, and S-028 samples are 8.36 Å, 8.35 Å, and 8.32 Å, respectively. The value of the lattice constant (a) for pure CoFe_2_O_4_ is 8.387 Å [36]. The precise lattice constant from Nelson-Riley function was estimated as 8.37 Å, 8.36 Å, 8.35 Å, for S-013, S-009, and S-028, respectively. A comparison of the lattice parameter obtained from Equation (2) with the lattice parameter from Equation (3) showed that the precise lattice parameters were broader. This variation of the lattice constant in S-009 and S-028 samples is the diffusion process of metallic ions (Cu^2+^, Co^2+^, Fe^3+^ ions), from a spinel to hematite structure during the sintering process (thermal energy) [57]. In the present series of ferrite samples, Cu^2+^ ions of greater ionic radius 0.73 Å substitute Co^2+^ ions of smaller ionic radius 0.65 Å, and the lattice parameter is expected to increase. However, it is not observed, and this may be because the contribution to the lattice parameter does not solely depend on the ionic radii but also on another interaction phenomenon, like the long-range attractive coulomb forces, bond length, etc., the calculation of which necessitates precise knowledge of the existence of any surface charge which needs further investigation [58]. Additionally, the results show that the lattice constant decreases with a decrement in the concentration of the ferrite phase or an increase of the hematite phase in S-009 28.4% and S-028 40.9% samples, respectively. The calculated lattice parameter is shown in Table 1.

The increase in particle size in the S-028 sample with the increase of hematite content may be explained by the fact that the hematite nano crystallites may agglomerate at the grain boundaries causing a steady increase in particle size [46].

Table 1 compares the results of microstrain ε, bulk density (d_bulk_), crystallite size L, lattice parameter (a), X-ray density (d_XRD_), porosity (P%), and the specific surface area S_SA_ of copper and cobalt substituted spinel ferrite powder particles synthesized at 950 °C.

X-ray density d_XRD_ and porosity P values of ferrite samples were computed by the following equations [46,59]:

(4) $$d_{XRD}=\frac{8M}{N_{A}a^{3}}$$

(5) $$P=\left(1-\frac{d_{bulk}}{d_{XRD}}\right)\times 100$$

where M is the molecular weight of the ferrites, (a) is the lattice parameter, (N_A_) is Avogadro’s number and 8 is the number of molecules per unit cell. The experimental (apparent) density d_bulk_ of the prepared ferrites was calculated using a method of geometrical shape by considering the cylindrical shape of the pellet sample where m, V, r, t are mass, volume, radius, and thickness of the pellet sample, respectively [60]. From Table 1, it was observed that the X-ray density for the S-013 ferrite had a lower value compared to the value obtained for the S-028 ferrite. This could be attributed to a higher Cu^2+^ content in the lattice structure of the ferrite. However, this behavior was supported by the effect of the propylene glycol cosolvent used for the synthesis of ferrites. Also, the bulk density, d_bulk_, had the same behavior. This fact suggests that the concentration of the hematite phase and the incorporation of Cu^2+^ ions in the lattice structure of the ferrite can create porosity [61]. Additionally, it was evidenced that the glycerin used as a cosolvent and standing time allows lower values of crystallite size, X-ray density, bulk density, and porosity. From Table 1, an increase in the concentration of Cu^2+^ and an increase in the value of the lattice parameter are related to a decrease in the value of X-ray density. In addition, an increase in the concentration of Cu^2+^ and a decrease in the value of the lattice parameter is related to an increase in the value of X-ray density. Due to the presence of pores, the X-ray density is higher than the bulk density. As the particles grow, the porosity decreases because of the cosolvent glycerin and the standing time. This fact suggests that the Cu doping may create the porosity in the S-028 ferrite as illustrated in Table 1

The specific surface area (SSA), defined as the total surface area per unit of mass, for adsorption and reactions on the surface of the material, can be calculated by taking advantage of the values of the structural parameters obtained from the XRD analysis, where it is taken that the value of the shape factor is equal to 6, as shown in the following equation: [62]

(6) $$S_{SA}=\frac{6}{L}{x\left(d_{XRD}\right)}^{-1}$$

The specific surface area values reported in Table 1, S_SA_ = 37.83 m^2^g^−1^ and S_SA_ = 38.81 m^2^g^−1^ of S-009 and S-013, respectively, indicate the increase of the S_SA_ value of ferrite with increasing Cu^2+^ ion content. However, the S_SA_ = 28.29 m^2^g^−1^ value was lower in the case of S-028 ferrite. This could be related to the effect of the cosolvent on the synthesis-increasing of crystallite size L of the S-028 ferrites studied. From Table 1 the specific surface area increases with decreasing particle size. The specific surface area is also increased if the particle has pores.

#### 3.1.2. Morphology and Surface Textural Properties

**Figure 3:**
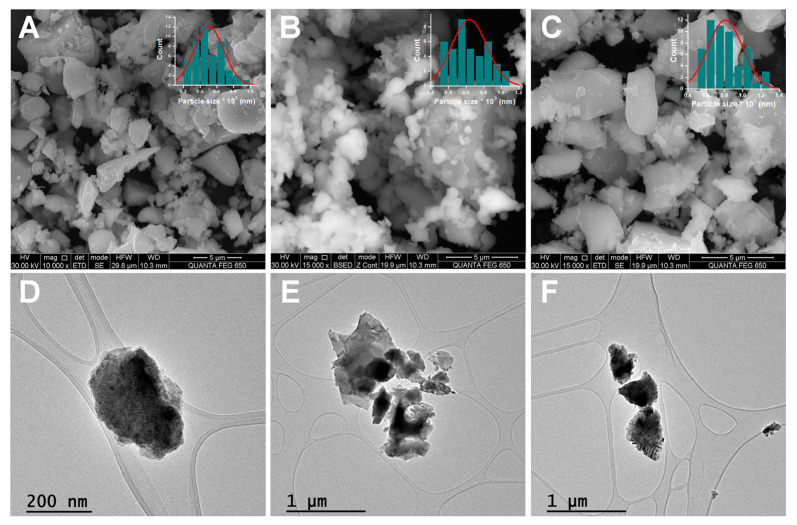
FESEM morphology (**A**) S-013, (**B**) S-028 (**C**) S-009 ferrites and TEM images (**D**) S-013, (**E**) S-028, and (**F**) S-009.

The morphology of the investigated ferrites was analyzed using FESEM, TEM micrographs revealed in Figure 3A–F. The synthesized spinel-type ferrite sample was chosen with a standing time of eight hours before calcination. It was possible to eliminate the impurity of the hematite phases present in the sample. Figure 3A–C showed a FESEM micrograph of grains of the particles, irregular shapes, and agglomeration of grains. Figure 3D–F showed TEM images of the agglomeration of grains where small nanoparticles are interlocked with large particles because of their magnetic nature. Subsequent acid washes helped to evaluate the composition of the spinel-type ferrite samples determined by atomic absorption spectroscopy. The composition corresponded to Cu_0.13_Co_0.87_Fe_2_O_4_, Cu_0.09_Co_0.91_Fe_2_O_4_, and Cu_0.28_Co_0.72_Fe_2_O_4_ labeled as samples S-013, S-009, and S-028, respectively.

From the TGA plots (Figure S2), it was evident that two stages can be distinguished: (i) a minimum decrease in weight that begins before 100 °C and culminates around 590 °C that can be assigned to the consumption of oxygen by the process of copper oxidation (see Figure S2A). The second weight loss is probably due to the combustion process and elimination of carbon. From the DSC curve, an endothermic process could be observed due to water loss (see Figure S2A).

The EDS analysis (Figure S3) of the synthesized LCF NPs showed the composition of the ferrite nanomaterial, the presence of copper, and no phases such as α-Fe_2_O_3_, CoO, CuO, Co_2_O_3_, or Co_3_O_4_ were found.

#### 3.1.3. Vibrational Properties

The FTIR spectra of copper and cobalt-substituted ferrite nanoparticles recorded in the 4000–450 cm^−1^ range, Figure S4. The band at 569 cm^−1^ is attributed to intrinsic stretching vibrations of the complex group in the tetrahedral sites of Fe^3+^-O^2−^, determined by the vibration of ions inside the crystal [63,64]. With the addition of Cu^2+^ ions, ν_1_ gradually changes randomly. This change in the values of ν_1_, can give an important indication of the substitution of cobalt ions with copper ions in the spinel structure, as reported in the literature [65]. In the case of a decrease in the value of the vibration frequency, the Cu–O bond length is extended in the octahedral-B sites and therefore, the binding energy is lower. In addition, if the absorption band of the samples is shifted to a higher wavenumber, it confirms the change in the interaction of the cosolvents on the nanoparticle surface [65]. The broad and weak absorption band around 980 cm^−1^ corresponds to the bending out of the O–H plane, of the water vapor adsorbed on the nanoparticles from the atmosphere [64] due to the porosity of the LCF nanoparticles, and to the adsorption and migration of iron ions and hydrated cobalt on the crystal surface [66].

**Figure 4:**
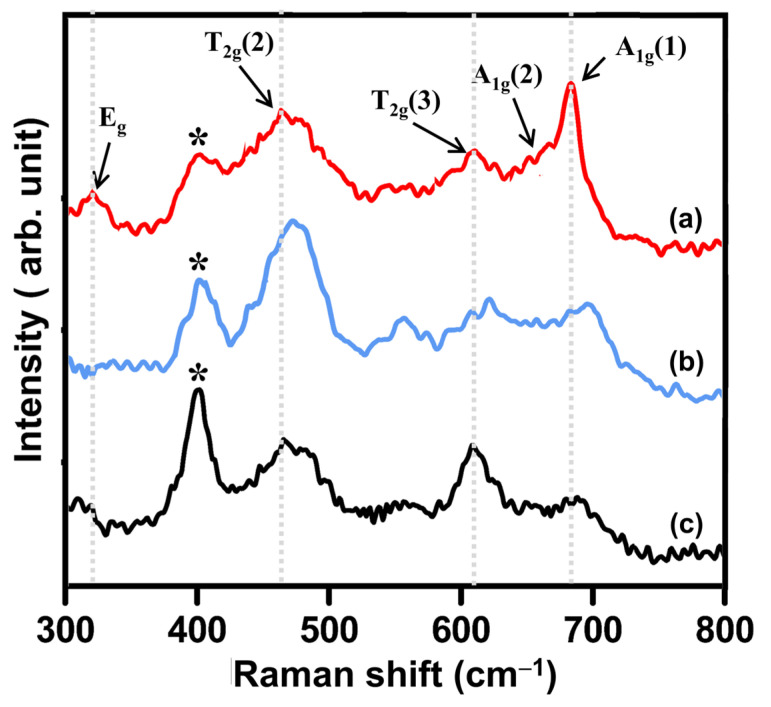
Raman spectra of LCF NPs. Effect of hydroxylated agents and standing time before calcination. (**a**) + glycerin and eight hour standing time S-013; (**b**) + glycerin, S-009; and (**c**) + propylene glycol S-028, respectively.* Raman signal of hematite phase at 400 cm^−1^.

Figure 4 shows the Raman spectra of the LCF NPs. The characteristic signs of the spinel-type ferrite species were evidenced. In Figure 4a the spectrum of S-013 ferrite, was shown, with wide bands at 325, 475, 571, 619 and a strong band at 681 cm^−1^. They are bands like the data reported by other authors [33].

In this species of iron oxide, iron ions were in both tetrahedral and octahedral sites, while cobalt only occupies octahedral sites. The difference in the ionic radii of these cobalt and iron ions caused the distances of the Fe-O and Co-O bonds to be distributed at these sites. The Raman bands at 681 and 619 cm^−1^ A_1g_(1) and A_1g_(2), respectively, correspond to the modes that reflect the stretching vibration of Fe^3+^ and O^2−^ ions at the octahedral sites (O site). The tetrahedral sites (T-site) reflect the vibrational modes, whose bands corresponded to 571, 475, and 325 cm^−1^ and were assigned to the T_2g_(3), T_2g_(2), and Eg modes, respectively [51]. From Figure 4a–c, a shift of the peaks towards a lower wavelength was evidenced, which is called a redshift. This resulted in a positive effect of the higher atomic mass of copper on cobalt. In addition to this, the random distribution of the charged species with a divalent and trivalent profile must have been affected by the changes in the mobility of the metal species ions from tetrahedral (A) to octahedral (B) sites and vice versa. In Figure 4, the decrease in the intensity of the hematite phase band in 400 cm^−1^ was also evidenced. This is due to the glycerin and longer standing time (eight hours) during the synthesis. These results are in correlation to those evidenced by a technical spectral XRD. According to these results, the samples S-013 and S-028 are studied below, evaluating their ability to sense acetaminophen.

### 3.2. Electrochemical Properties of LCF NPs

#### Electrochemical Activity Measurements

Initially, the electrochemical behavior of the LCF nanoparticle samples S-013 and S-028 was studied using cyclic voltammetry and differential pulse voltammetry DPV in a 0.1 M phosphate-buffered saline (PBS) solution.

In a 20 µM acetaminophen AC solution, the modification of the GCE electrode with the nanoparticle sample S-013 demonstrated a higher peak current compared to the bare electrode and the electrode modified with the nanoparticle sample S-028 (see Figure S5). This suggests that this nanomaterial possesses a slightly larger active surface area and higher conductivity. Additionally, CVs indicated that due to these properties, the electrochemical response of the S-013/GCE electrode was better (see Figure S6).

Modified and bare electrodes were also studied to determine the electron transfer rate using a 2.5 mM solution of [Fe(CN)_6_]^3−/4−^ in 0.1 M KCl. The anodic and cathodic peak potential values were determined from the voltammograms obtained using the modified S-013/GCE, S-028/GCE, and bare electrodes, respectively. The separation between the anodic and cathodic peak potentials (ΔEp) is related to the electron transfer rate. The ΔEp values followed the order 154 < 256 < 340 mV for S-013/GCE, bare GCE, and S-028/GCE electrode, respectively. This indicates that the S-013/GCE electrode has a faster rate of electron transfer (see Figure S7A). Furthermore, the strong electrocatalytic activity of the modified S-013/GCE electrode was evident in the improvement of both anodic and cathodic signals.

Through cyclic voltammetry, a study using the Randles-Sevcik equation (Equation (S1)) revealed a clear relationship between the current peak I_p_ and the square root of the scan rate v^1/2^. From the slope value of I_p_ vs. v^1/2^, the electrochemical active surface area (EASA) was calculated, and its values were 0.96, 0.62, and 0.45 cm^2^, for the S-013/GCE, S-028/GCE, and GCE electrodes, respectively. In this case, the active surface area of S-013/GCE is approximately 2.0 times greater than the bare electrode area, showing that S-013/GCE is most appropriate for electrochemical sensing applications.

The electron transport of the modified electrodes was examined using electrochemical impedance spectroscopy in a solution of 2.5 mM [Fe(CN)_6_]^3−/4−^ in 0.1 M KCl. The charge transfer resistance (R_ct_) of the electrodes S-013/GCE, S-028/GCE, and bare GCE were evaluated using the Nyquist plot (Figure S7B). The R_ct_ on the S-028/GCE, bare GCE, and S-013/GCE are approximately 45.4, 19.2, and 12.0 kΩ, respectively. In the case of the modified electrode S-013/GCE, the diameter of the semicircle shows that R_ct_ decreased. EIS measurements showed that S-013/GCE was more electroactive and conductive than the bare electrode. Equation (S2) was used to determine the standard heterogeneous rate constant and whose values were obtained. 9.24 × 10^−6^, 8.94 × 10^−6^, and 5.21 × 10^−6^ cm s^−1^, for the S-013/GCE, S-028/GCE, and bare electrode, respectively. Therefore, improved electron transport kinetics were deduced in the S-013/GCE modified electrode.

### 3.3. Improving Conditions for the Development of Sensitive and Selective LCF NPs/GCE Electrodes

The Influence of Chemical and Physical PropertiesParameters of interest were studied to achieve optimal working conditions which can influence the sensitivity and electroactivity of the electrodes modified with LCF NPs. The amount of surface modifying nanomaterial, concentration of nanomaterial, temperature effect, pH, and mechanical stirring effect of the electrodes modified with LCF NPs were evaluated.

From Figure S8, an increase in the acidity of the electrolytic medium causes an increase in the oxidation current value of AC. As the acidity of the medium decreases (values above pH 2.5), the oxidation current value decreases on the surface of the LCF NPs/GCE electrode. Such behavior suggests that chemical reactions follow the electrochemical mechanism, wherein the buffer molecules and the oxidized form of the electrode may react [66,67]. It is assumed that LCF NPs exhibit electrocatalytic activity as ferrite (Fe_3_O_4_) involving the iron phosphate system according to the reaction [50,66,67].

(7) $${Cu}_{x}{Co}_{\left(1-x\right)}{Fe}_{2}O_{4}+e^{-}\to {Fe}^{2+}\left[{Cu}_{x}{Co}_{\left(1-x\right)}FeO_{4}\right]$$

(8) $${Fe}^{2+}\left[{Cu}_{x}{Co}_{\left(1-x\right)}FeO_{4}\right]+{H_{2}PO}_{4}^{-}\to FeH{PO}_{4}\left[{Cu}_{x}{Co}_{\left(1-x\right)}FeO_{4}\right]+H^{+}$$

(9) $$FeH{PO}_{4}\left[{Cu}_{x}{Co}_{\left(1-x\right)}FeO_{4}\right]+H^{+}\to {Cu}_{x}{Co}_{\left(1-x\right)}{Fe}_{2}O_{4}{+e^{-}+H}_{2}{PO}_{4}^{-}$$

Furthermore, the oxidation potential of AC shifted to lower values, as the pH value increased (insert in Figure S8): 0.049 V at the surface of the S-013/GCE, 0.059 V at the surface of the bare GCE, and 0.028 V at the surface of S-028/GCE. An equivalent number of charged subatomic particles in the redox system are related to slope values close to the Nernstian (0.059 V/pH) [67]. From the results obtained, it was deduced that pH 2.5 was better for the AC oxidation.

To determine the optimal concentration of LCF NPs, doses in the range of 0.2 to 2.0 mg mL^−1^ were used to achieve the highest current efficiency (see Figure S9). The current percentages were improved as the amount of LCF NPs increased and was related to a greater number of available functional adsorption sites and a greater surface area. At higher concentrations of LCF NPs (over 1.0 mg mL^−1^), there was a drop in the peak current intensity, which could be attributed to the overlap or aggregation of accessible binding sites and the reduction of the total surface area of the available adsorbent.

The influence of the nanocomposite’s quantity in a 5.0 μM AC solution was then examined using differential pulse voltammetry (DPV). The suspensions of various concentrations of LCF NPs were applied to the GCE electrode to alter its surface (Figure S10). In separate determinations, between 2.0 and 14.0 µL of the LCF NPs suspension were dropped onto the electrode surface. Subsequently, each suspension addition was carefully dried at room temperature. As the amount of suspension was increased, it was revealed that the intensity of the peak current Ip was better (I_p_ = 1.07 µA) with 8.0 µL of the LCF NPs suspension. At higher suspension amounts, a notable decrease in sensitivity, poor diffusion, and adhesion were detected due to the dense layer of the LCF NPs on the electrode surface. As a result, the optimal volume was 8.0 µL of the suspension LCF NPs 1.0 mg mL^−1^. In Figures S11 and S12, the optimal conditions of electrolyte temperature and stirring speed were found to be 27.5 °C and 250 rpm, respectively.

**Figure 5:**
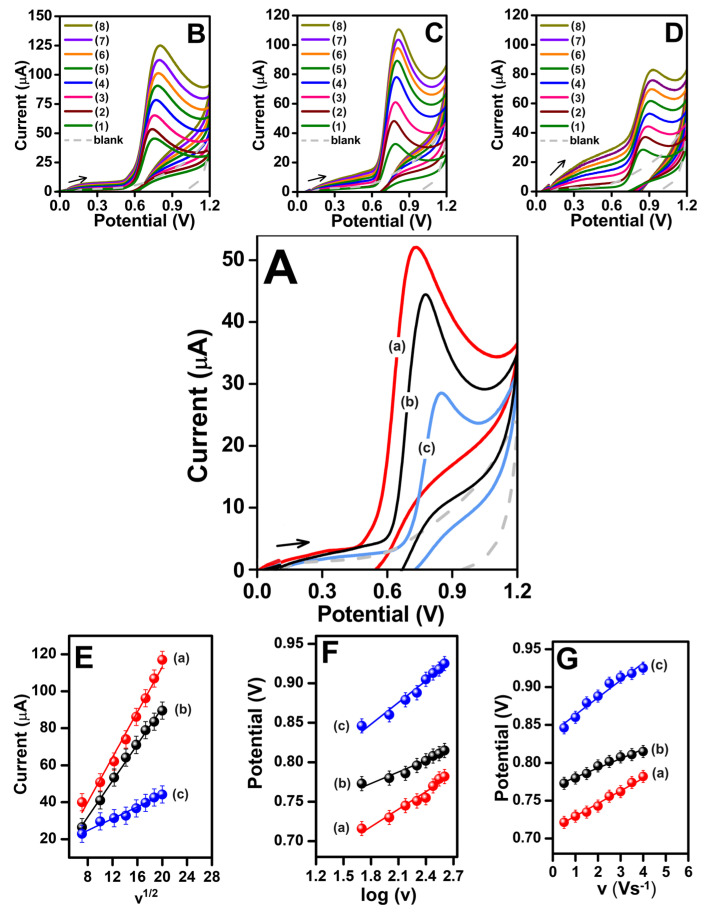
(**A**) Cyclic voltammograms (CVs) of 2.5 × 10^−3^ mol L^−1^ AC in 0.1M PBS (pH = 2.5), scan rate: v = 0.1 Vs^−1^. (a) S-013/GCE, (b) GCE bare, and (c) S-028/GCE electrode, respectively. The dashed line represents the absence of AC in solution on modified electrode. (**B**) S-013/GCE, (**C**) GCE and (**D**) S-028/GCE CVs at different scan rates (1) 0.05, (2) 0.1, (3) 0.15, (4) 0.20, (5) 0.25, (6) 0.30, (7) 0.35, and (8) 0.40 Vs^−1^. (**E**) peak currents (μA) versus square of scan rate (v^1/2^), (a) I_p_ = 6.08 v^1/2^ − 8.54, r^2^ = 0.964; (b) I_p_ = 4.91 v^1/2^ − 7.31, r^2^ = 0.992, and (c) I_p_ = 1.60 v^1/2^ − 11.91, r^2^ = 0.986. (**F**) Linear plots of E_p_ vs. logarithm (v), and (**G**) E_p_ vs. v.

Figure 5 shows the comparative CVs of 2.5 mM AC at 0.1 M PBS buffer, pH 2.5, on LCF NPs (bare and modified electrodes), and a scan rate 0.1 Vs^−1^. From Figure 5A, it was evidenced that the oxidation of AC on the S-013/GCE electrode yielded a maximum current response I_p_ = 48.56 µA while low values of I_p_ = 36.47 µA and 25.64 µA for the bare electrode and S-028/GCE electrode were obtained. These results led to better electrical conductivity and higher mass transport, which may be due to the presence of LCF NPs nanoparticles [68].

In Figure 5A, the anodic peak potential at the modified S-013/GCE electrode was less positive than that at the bare electrode and S.028/GCE electrode, respectively. These facts indicated that the synthesized S-013 nanoparticles effectively catalyzed the electrochemical oxidation of AC [69]. Regarding the LCF NPs in this study, the greatest effect occurred when the S-013 nanoparticles synthesized through combustion using glycerin and a standing time of 8 h were used.

Figure 5B–D exhibits CVs of 2.5 × 10^−3^ mol L^−1^ AC in 0.1M PBS (pH = 2.5) at a scan rate from 50 to 400 mV s^−1^ of the S-013/GCE, GCE bare and S-028/GCE electrode, respectively. A systematic relation in I_p_ vs. v and E_p_ vs. Log v, was observed [70].

The curve I_p_ vs. v^1/2^ are shown in Figure 5E. The equations and the linear relations between the current and the square root of the scan rate v^1/2^ at the LCF NPs/GCE suggest that the electrooxidation of AC is under diffusion control. Figure 5F exhibits a linear relationship E_p_ vs. log v for AC, E_p_ = 0.074 log(v) + 0.588 r^2^ = 0.898 for S-013/GCE, E_p_ = 0.049 log(v) + 0.674 r^2^ = 0.895 for GCE bare and E_p_ = 0.092 log(v) + 0.682 r^2^ = 0.931 for the S-028/GCE electrode. The E_p_ value was displaced towards more positive values which suggests that AC oxidation is irreversible in nature [71]

Lower peak potential values were evident from Figure 5F using the S-013/GCE electrode compared to those obtained for the GCE bare and S-028/GCE electrode. At each modified electrode, the behavior of the anodic peak potential value versus the scanning rate (Figure 5G) revealed that the electrochemical process for the anodic oxidation of AC was irreversible. This could be attributed to the superior catalytic capacity of LCF NPs [72].

In Laviron’s theory, the peak potential E_p_ for an irreversible electron transfer can be expressed by the following equation [73].

(10) $$E_{pa}=E^{0}+\left(\frac{2.30RT}{\alpha nF}\right)log\left(\frac{RTk^{0}}{\alpha nF}\right)+\left(\frac{2.30RT}{\alpha nF}\right)log(v)$$

where α is the transfer coefficient, k^0^ the standard heterogeneous rate constant of the reaction, n the number of electrons transferred, v the scan rate and E^0^ is the formal redox potential, F = 96,485 C mol^−1^, T = 298 K, R = 8.314 J K^−1^ mol^−1^. Therefore, the value of αn and k^0^ can be easily calculated from the slope and intercept from the E_p_ versus log v plot. E^0^ was obtained from the intercept, extrapolating to the vertical axis at v = 0 the curve Ep versus v [72].

The E^0^, k^0^ and αn values were obtained 0.713 V, 0.63 s^−1^, and 0.80 for the S-013/GCE electrode and 0.768 V, 0.59 s^−1^, and 1.20 for the GCE electrode 0.840 V, 0.52 s^−1^, 0.62, respectively [74]. It can be observed that the k^0^ and αn values are higher for the S-013/GCE electrode because the results are associated to the major electrocatalytic activity in the mixed oxide samples [75]. The (α) value was calculated according to the Bard and Faulkner equation [72].

(11) $$\alpha =\frac{47.7}{E_{p}-E_{\frac{p}{2}}}$$

where E_p/2_ is the potential when the current is at half the peak value. From this, the value of (α) was calculated to be 0.43, 0.54, 0.42, and the number of electrons transferred during AC electrooxidation were 1.86, 2.22, and 1.47 in the S-013/GCE, bare GCE, and S-028/GCE electrodes, respectively. The results confirmed that two electrons and different amounts of protons are transferred during AC electrooxidation. The AC has hydroxyl and amine functional groups in its structure, and its redox behavior can be affected by the acidity of the supporting electrolyte PBS buffer.

**Scheme 1:**
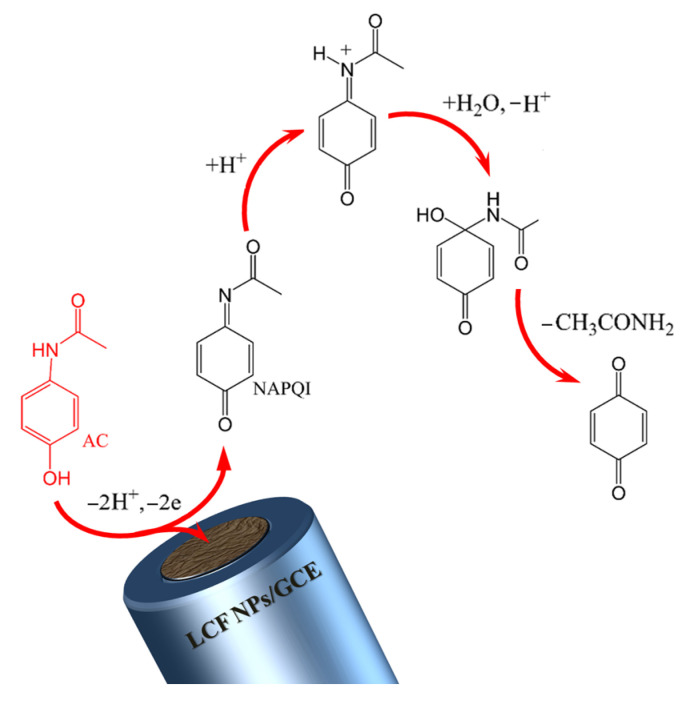
The mechanism of possible oxidation of AC.

Taking these results into account, as well as the use of the slope of E_p_ vs. pH, and using Laviron’s, a possible redox reaction sequence for the AC was shown in Scheme 1, in which two electrons transfer and the inclusion of the protons was involved in the oxidation of acetaminophen (AC) to N-acetyl-p-benzoquinone-imine (NAPQI).

### 3.4. AC Determination on a Modified LCF NPs/GCE Electrode

#### 3.4.1. Differential-Pulse Voltammetric DPV Determination of AC at the Modified LCF NPs/GCE Electrode

**Figure 6:**
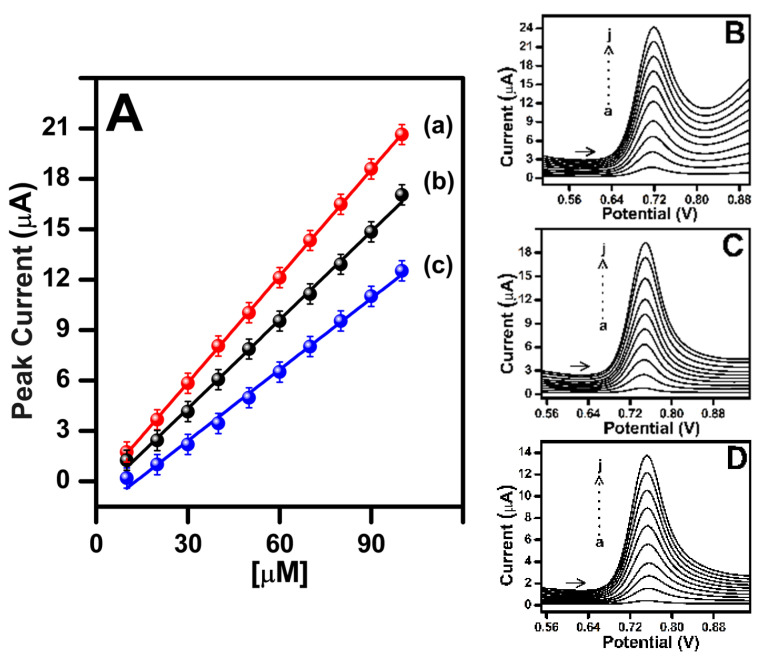
(**A**) Plot of oxidative peak current versus concentration of AC respectively at (a) S-013/GCE, (b) S-009/GCE, and (c) GCE bare electrode in the concentration range of (a–j: 10, 20, 30, 40, 50, 60, 70, 80, 90 and 100 μM). (**B**–**D**) Differential pulse voltammograms of AC with increasing concentrations at (a), (b), and (c) respectively.

To develop a voltammetric method to determine AC, we selected the DPV mode, because it is more sensitive than the CV mode. According to the results obtained, it was possible to apply this technique to the determination of AC. Figure 6A presents a plot of the oxidative peak current versus concentration of the AC, respectively, at the (a) 579 S-013/GCE, (b) GCE bare, and (c) S-028/GCE electrode in the concentration range of (a–j: 10, 20, 30, 40, 50, 60, 70, 80, 90, and 100 μM).

The DPV voltammograms, which reported current peaks and were directly correlated with the AC concentration, were acquired using a PBS buffer with a pH of 2.5 as a supporting electrolyte [76]. Figure 6B–D presents the DPVs voltammograms of AC with increasing concentrations at the (a) S-013/GCE, (b) GCE bare, and (c) S-028/GCE electrode. The maximum height of the peak was measured from the baseline to its maximum to establish the peak current. As mentioned in the manuscript text below, the measurements were repeated. The increase in peak current with the rising AC concentration in the voltammogram above is due to the presence of more ions in the solution, which allows the electrons to flow more easily [77]. The oxidative peak current showed a linear dependence on the AC concentration (Figure 6A) in the range 10–100 μM with I_p_ (μA) = 0.2113 [AC] (μM) − 0.4661 (R^2^ = 0.9998) I_p_ = 0.1640 [AC] − 1.0450 (R^2^ = 0.9994) and I_p_ = 0.1412 [AC] − 1.8233 (R^2^ = 0.9957) at the (a) S-013/GCE, (b) GCE bare, and (c) S-028/GCE electrode (Figure 6A). The reduced slope value is probably due to a kinetic limitation during the electrochemical oxidation at the S-028/GCE electrode. The limit of detection (LOD) and the limits of quantitation (LOQ) were calculated using the LOD = 3 s/m and LOQ = 10 s/m, where s is the standard deviation of the blank solution (ten runs) and m is the slope of the related calibration curves. The relative standard deviation (RSD) was calculated by the standard deviation divided by the mean of 10 repeated measurements by 100. Therefore, the limit of detection (LOD) was calculated as (99.4, 128, and 138 nM), the limit of quantification (LOQ) was found to be (331, 421, and 460 nM) and the RSD 3.31%, 4.27%, and 4.60% for the S-013/GCE, GCE bare, and S-028/GCE electrode, respectively. The superior electrocatalytic activity of the S-013/GCE electrode correlates very well with its R_ct_ resistance found using the EIS technique, which defines the electron transfer capacity of the S-013 nanoparticles. The results showed that the modified S-013/GCE electrode has a lower detection limit with adequate sensitivity. The detection limit obtained for S-013/GCE in the present study (99.4 nM) is lower than the other electrodes studied here, including most of the modified electrodes shown in Table S1. The influence of the possible interferences on the electrochemical redox of AC at the S-013/GCE electrode was investigated with the presence of glucose, ascorbic acid, uric acid, and of the inorganic ions such as K^+^, Na^+^, Ca^2+^, and Mg^2+^. These were chosen because they are normally found in food and in the environment [78], and because it has been reported that metal ions and inorganic salts could interfere with the biosensors based on nanomaterials [79]. The signals are almost unchanged, which means that all these inorganic ions have no obvious interference with the detection of AC (Figure S13A). As shown in Table S2, 100-fold concentrations of glucose, ascorbic acid, uric acid, and of the inorganic ions such as K^+^, Na^+^, Ca^2+^, and Mg^2+^ almost do not affect AC’s current response. The results indicate the electrochemical sensor based on S-013/GCE exhibits good selectivity towards the AC.

**Figure 7:**
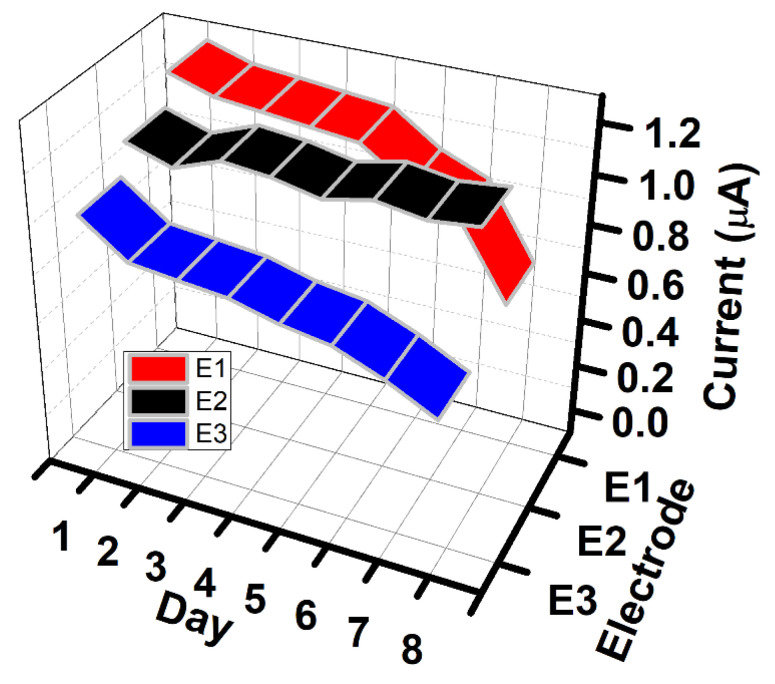
Long-term response stability of three independent electrodes E1 = S-0.13/GCE, E2 = GCE, and E3 = S-028/GCE subjected to 10.0 µM AC in 0.1 M PBS buffer (pH 2.5). The peak current Ip from DPV voltammograms was monitored every 24 h for eight days. DPV settings (a pulse times of 40 ms, 0.3 ≤ Epa ≤ 1.2 V at a step potential of 4 mV, a scan rate of 8 mVs^−1^, and pulse amplitudes of 80 mV).

In addition, to assess the stability of the long-term response [80], continuous measurements were performed with the aid of DPV pulse differential voltammetry, and glass vials with parafilm-wrapped caps were used to preserve the S-013/GCE, GCE, and S-028/GCE in 0.1 M PBS (pH 2.5) at 4 °C. After every 24 h (1 day), electrochemical oxidation current measurements of 10 µM AC mixed with a PBS buffer (pH 2.5) were performed. Figure 7 shows that the anodic peak current measured by the DPV technique had a slight decrease of 6.6% from its initial response for at least 4 days or four consecutive repetitions of voltammetric measurements. A similar behavior was obtained with the E3 = S-028/GCE electrode, but with a greater decay in the intensity of the anodic peak current (Figure 7). The DPV signal obtained using the E2 = GCE bare electrode was stable and of lower value. After five repetitions of DPV measurements, the signal of the E1 = S-013/GCE electrode decreased even more, up to 48.8%, from its initial response compared to the stability of the GCE electrode and the result shown by the electrode modified with S-028 nanoparticles. These results indicate that the S-013/GCE electrode can be reused for at least 4 days, a longer time compared to the S-028/GCE electrode used.

#### 3.4.2. Analysis of Real Urine Samples

**Figure 8:**
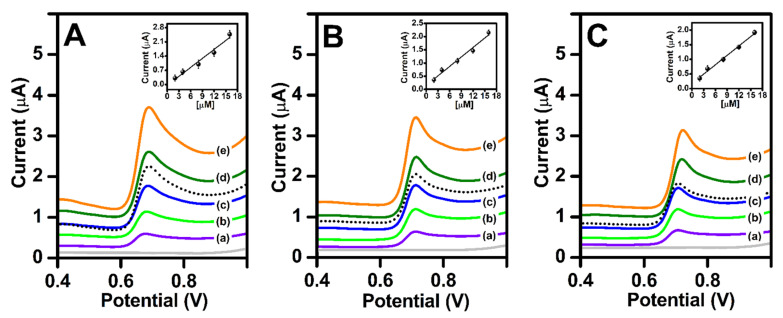
Differential pulse voltammograms of acetaminophen AC, prepared in urine media and five samples with additions of (a) 2.0, (b) 4.0 (c) 8.0, (d) 12.0, and (e) 16.0 µM, respectively. Dot line 10.0 µL of real urine sample 4 h after consuming the AC drug. The insets represent corresponding calibration dependences. (**A**) S-013/GCE; I_p_ = 0.147 [AC] − 0.038, r^2^ = 0.9654, (**B**) GCE bare; I_p_ = 0.120 [AC] + 0.1513, r^2^ = 0.9756, and (**C**) S-028/GCE; I_p_ = 0.107 [AC] + 0.174, r^2^ = 0.9894 electrodes, respectively. The gray line indicates the absence of AC.

For testing the possibility of the determination of AC in human urine samples, the method of the calibration curve was used. Calibration curves and ranges of determination for the analyzed drug in model samples of human urine gave a linear response in the concentration ranges of 2–16 µM (Figure 8). According to Figure 8, the behavior of the peak potentials and even more of the peak currents remain relatively consistent with the results obtained up to this point in this work. From a patient with no history of diseases 4 h after consumption of AC, a real human urine sample was used to determine the excreted AC content. The device was found to respond to the detection of AC in the actual sample.

The calibration curves show a linear response over the whole range of concentrations used in the assay procedure and their parameters are summarized in Table 2. No interference of the matrix components was observed even without any sample pretreatment. Table 2 shows the results of the five investigated urine samples with an AC standard. Real urine sample 6 was analyzed after 4 h of the AC drug consumption.

The calibration chart was used in the analysis of enriched AC in the urine samples with recovery ranges from 96.5 to 101.0% and 88.01 to 90.50% for the S-013/GCE and the GCE bare electrodes, respectively, indicating the outstanding analytical performance of the modified electrode. Table 2 demonstrates that there is a modest difference in the findings obtained between the modified S-013/GCE electrode and the GCE electrode when determining the amount of AC in urine samples. The disparity is not very significant. Nevertheless, based on the amount of AC that may be expelled, the modified S-013/GCE electrode competes with the GCE electrode to enable the detection of the AC drug concentrations in the urine. Other studies with 90% average excretion of AC in urine after 4 h have been reported earlier [81,82,83,84,85,86] which strengthens the confirmation of our results which showed that the surface-modified electrode served for the determination of the AC drug in human urine samples.

## 4. Conclusions

LCF NPs were synthesized using a modified combustion approach. The effect of the cosolvent was evaluated and it was concluded that eco-friendly compounds such as glycerin could be useful for the synthesis of LCF NPs nanoparticles. However, rest periods were necessary during the synthesis. For the evaluation of the electrodes modified with LCF NPs, the potential scanning window conditions and acidity level necessary to obtain the most intense current signal were first optimized, achieving the best CV response in a 2.5 mmol L^−1^ PBS buffer (pH 2.5) and scanning potential from 0.2 to 1.2 V. The AC oxidation was indicated by the electroactivity of the S-013/GCE and the GCE bare electrodes. The nanoparticles improved the electron transfer rate, and the AC oxidation current on the surfaces was not significantly improved. The analytical performance of the different electrodes (S-013/GCE, GCE and S-028/GCE) was compared for the determination of the AC by DPV. The S-013/GCE sensor exhibited only slightly superior voltammetric behavior. The modified S-013/GCE electrode enhanced oxidation currents of AC noticeably. However, the S-013/GCE was selected as the optimal sensor for the determination of the AC analyte at low concentrations. In the PBS buffer (pH 2.5), the noticeable characteristics of the modified S-013/GCE electrode were good recoveries (95.5 to 101%) and had a high reproducibility (RSD of 0.69 to 4.66%) accompanied by a facile preparation. A linearity range quantification and detection limit of 10.0 to 100.0 µM, 331 nM and 99.4 nM, were observed for the S-013/GCE, respectively. Furthermore, no interference was seen from the typical species existing glucose, ascorbic acid, uric acid, and inorganic ions which suggest a selectivity of the modified electrode for an accurate analysis of the AC in different matrices and biological samples. The analysis of urine samples enriched with CA showed that the recovery varies from 96.5 to 101.0% and from 88.01 to 90.50% for the S-013/GCE and GCE bare electrodes, respectively. This indicates an outstanding analytical performance of the modified electrode and strengthens the confirmation that the S-013/GCE surface-modified electrode served for the determination of the AC drug in a human urine sample.

## Figures and Tables

**Table 1:** The microstrain ε, crystallite size L, X-ray density (d_XRD_), lattice parameter bulk density (d_bulk_), porosity (P%), and specific surface area S_SA_ of copper and cobalt substituted spinel ferrite powders particles synthesized at 950 °C.

Sample	L (nm)	^†^ ε (×10^−4^)	^ψ^ a (Å)	d_XRD_ (g cm^−3^)	^δ^ d_bulk_ (g cm^−3^)	* P (%)	^ϕ^ S_SA_ (m^2^ g^−1^)
S-009	29.7	−4.7	^‡^ (8.36) 8.35	(5.34) 5.36	5.19	2.81	(37.83) 37.69
S-013	29.0	−3.7	(8.37) 8.36	(5.33) 5.35	5.20	2.44	(38.81) 38.67
S-028	39.5	+2.3	(8.35) 8.32	(5.37) 5.43	5.21	2.98	(28.29) 27.97

**^†^** Determinate from Williamson–Hall method W-H, Equation (1); **^ψ^** Found from Bragg’s, Equation (2); ^‡^ obtained from Nelson Riley model, Equation (3); **^δ^** calculated using method of geometrical shape by considering the cylindrical shape of pellet sample where m, V, r, t are mass, volume, radius, and thickness of the pellet sample, respectively; * discovered using d_XRD_ data in parenthesis and Equation (5); ^ϕ^ estimated from Equation (6), using data L and d_XRD_ in parentheses.

**Table 2:** Determination of AC in urine samples.

Urine	Added (μM)	^(a)^ Detected (µM)		Recovery		SD ± RSD (%)	
		S-013/GCE	GCE	S-013/GCE	GCE	S-013/GCE	GCE
**Sample 1**	1.0	1.01	0.88	101.0	88.01	0.04 ± 3.96	0.04 ± 4.54
**Sample 2**	2.0	1.93	1.78	96.50	89.03	0.09 ± 4.66	0.06 ± 3.37
**Sample 3**	4.0	3.94	3.26	98.50	81.50	0.13 ± 3.28	0.09 ± 2.76
**Sample 4**	8.0	7.98	7.14	99.75	89.25	0.15 ± 1.88	0.12 ± 1.68
**Sample 5**	16.0	15.96	14.48	99.75	90.50	0.11 ± 0.69	0.21 ± 1.45
^(b)^ **Sample 6**	-	10.75	8.63	-	-	0.39 ± 3.68	0.38 ± 4.40
	15	24.81	22.38	99.24	89.52	0.27 ± 1.09	0.37 ± 1.65

^(a)^ Average of three replicates, ^(b)^ Real urine sample 4 h after consuming Ac drug.

## Data Availability

Data available in manuscript and the Supplementary Material.

## Supplementary Materials

The following supporting information can be downloaded at: https://www.mdpi.com/article/10.3390/bios13120997/s1, References [87,88,89,90,91,92,93,94,95,96,97] are cited in the Supplementary Materials. Figure S1: Representation of the experimental sequence of preparation and monitoring of electrochemical signals during acetaminophen sensing, using LCF NPs spinel-type. Figure S2: TGA spectra of the LCF NPs (a) + glycerin and eight hour standing time S-013; (b) + glycerin, S-009; and (c) + propylene glycol S-028, respectively. Figure S3: SEM images and quantitative results obtained from EDS analysis of synthesized LCF NPs. A. S-013; B. S-009 and C. S-028 samples, respectively Figure S4: FTIR spectra of LCF NPs in the frequency range of (A) 3600–500 cm^−1^, (B) 500–800 cm^−1^. Effect of hydroxylated agents and standing time before calcination. (a) + glycerin, S-009; (b) + glycerin and eight hour standing time S-013; and (c) + propylene glycol S-028, respectively. Figure S5: DPVs of 20.0 µM AC in 0.1 M PBS at pH2.5. The S-013/GCE electrode (red), the GCE bare electrode (blue), and the S-028/GCE electrode (black), respectively. Figure S6: CVs of the bare electrode, the S-013/GCE, GCE and S-028/GCE electrode in the absence of AC in 0.1M PBS, *v*: 0.05 Vs^−1^. Figure S7: A. Cyclic voltammograms CV at *v*: 0.1Vs^−1^, and B. Electrochemical Impedance Spectroscopy EIS, respectively, of 2.5 mM [Fe(CN)_6_]^3−/4−^ 0.1 M KCl, v: 0.1Vs^−1^ (a) S-013/GCE, (b) GCE bare electrode, (c) S-028/GCE electrode. Figure S8: Effect of pH values on the peak potential (dash line) and peak current (solid line) of AC on the LCF NPs/GCE electrodes at various pH values, *v*: 0.05 Vs^−1^. A. S-013/GCE, B. GCE bare, and C. S-028/GCE electrodes. Insert: cyclic voltammograms of solution containing 2.5 mM AC + PBS buffer in the pH range of 2.5–8.5 at the LCF NPs/GCE. Figure S9: Effect of S-013 nanoparticles concentration on AC response using S-013/GCE electrode with 10.0 µM AC in 0.1 M PBS. Insert show additions of 0.2, 0.3, 0.5, 1.0, 1.5, 1.8, and 2.0 mg mL^−1^ of S-013 nanoparticles, respectively. Figure S10: Effect of S-013 amount on AC response using the S-013/GCE electrode, with 10.0 µM AC in 0.1 M PBS. Insert show additions of 2.0, 4.0, 6.0, 8.0, 10.0, 12.0, and 14.0 µL of S-013 nanoparticles, respectively. Figure S11: DPV signals from 10 µM AC in 0.1 M PBS. Effect of solution temperature. Figure S12: DPV signals from 10 µM AC in 0.1 M PBS. Effect of solution stirring speeds. Table S1: Comparison of the electrocatalytic performance of different modified electrodes for the determination of AC. Figure S13: A. DPVs of 5 μM AC in the presence of various 100-fold (a) K^+^, (b) Ca^2+^, (c) Na^+^, (d) Mg^2+^, (e) ascorbic acid, (f) uric acid, and (g) glucose. B. The reproducibility curves of 10 μM AC at six different S-013/GCE electrodes, E1 to E6, respectively. Table S2: The effect of different interferences at S-013/GCE in the determination of AC.
