# Supplementary material for: A Facile Glycerol-Assisted Synthesis of Low-Cu2+-Doped CoFe2O4 for Electrochemical Sensing of Acetaminophen
Source: Biosensors (Basel). 2023 Nov 23;13(12):997. doi: 10.3390/bios13120997 (PMC10741980; doi:10.3390/bios13120997)
Supplement: Supplementary file 1 [file biosensors-13-00997-s001.zip › biosensors-2658556-supplementary.pdf]

A facile glycerol-assisted synthesis of low-Cu^{2+}-doped $CoFe_2O_4$ for electrochemical sensing of acetaminophen

José Guillermo Alfonso-González [1], Claudia Patricia Granja-Banguera [1,+], Jimmy Alexander Morales-Morales [1,*] and Andrés Dector [2,+]

[1] Grupo de Investigación en Química y Biotecnologia (QUIBIO), Facultad de Ciencias Básicas, Campus Pampalinda, Universidad Santiago de Cali, Cali 760035, Colombia; jose.alfonso00@usc.edu.co (J.G.A.-G.); claudia.granja00@usc.edu.co (C.P.G.-B.)
[2] CONAHCYT, (Consejo Nacional de Humanidades, Ciencias y Tecnologías), Universidad Tecnológica de San Juan del Río, San Juan del Río, 76800, Querétaro, México; adector@conahcyt.mx (A.D.)
* Correspondence: jimmy.morales00@usc.edu.co
† These authors contributed equally to this work.

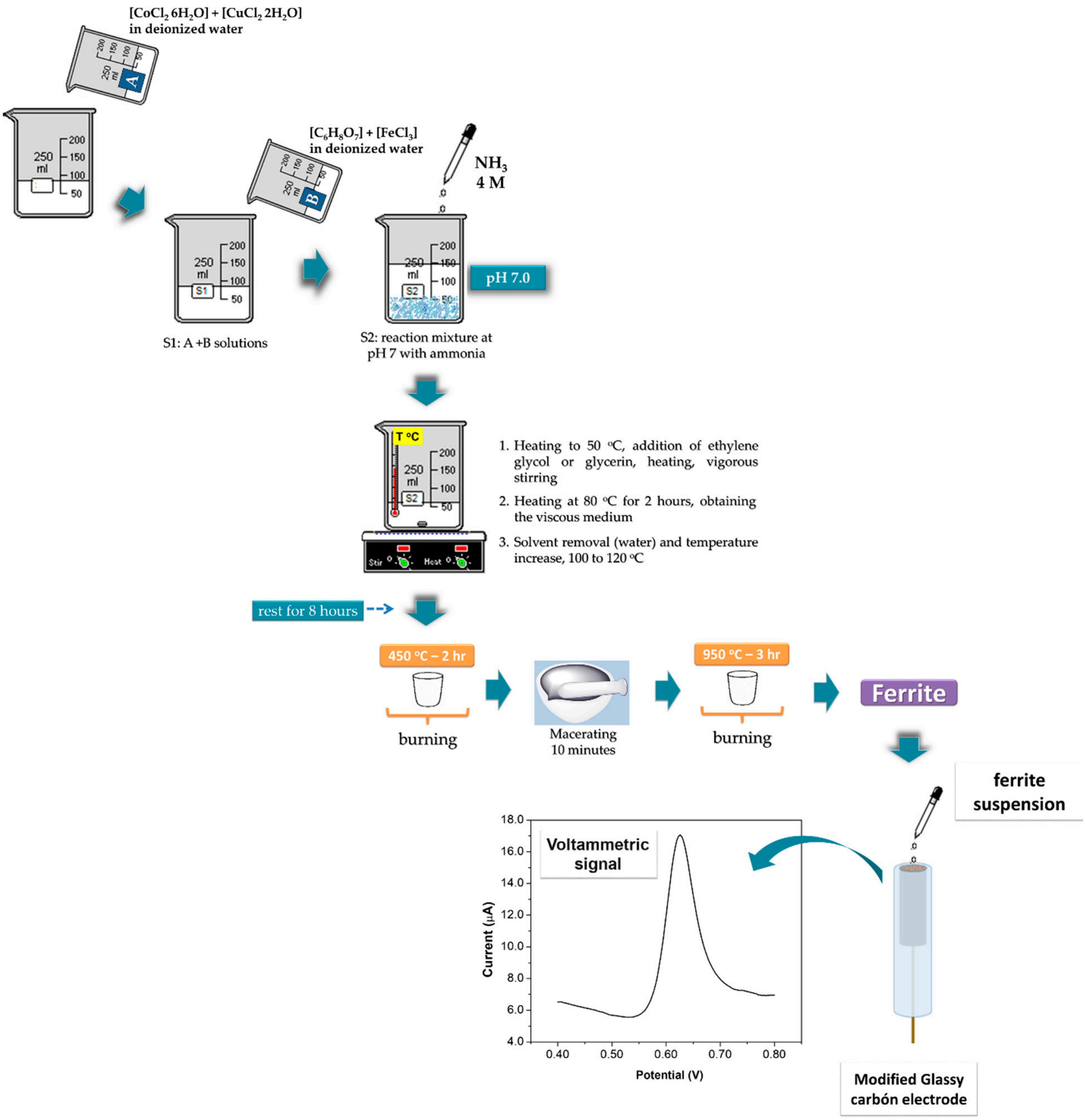

Figure S1. Representation of the experimental sequence of preparation and monitoring of electrochemical signals during acetaminophen sensing, using LCF NPs spinel-type.

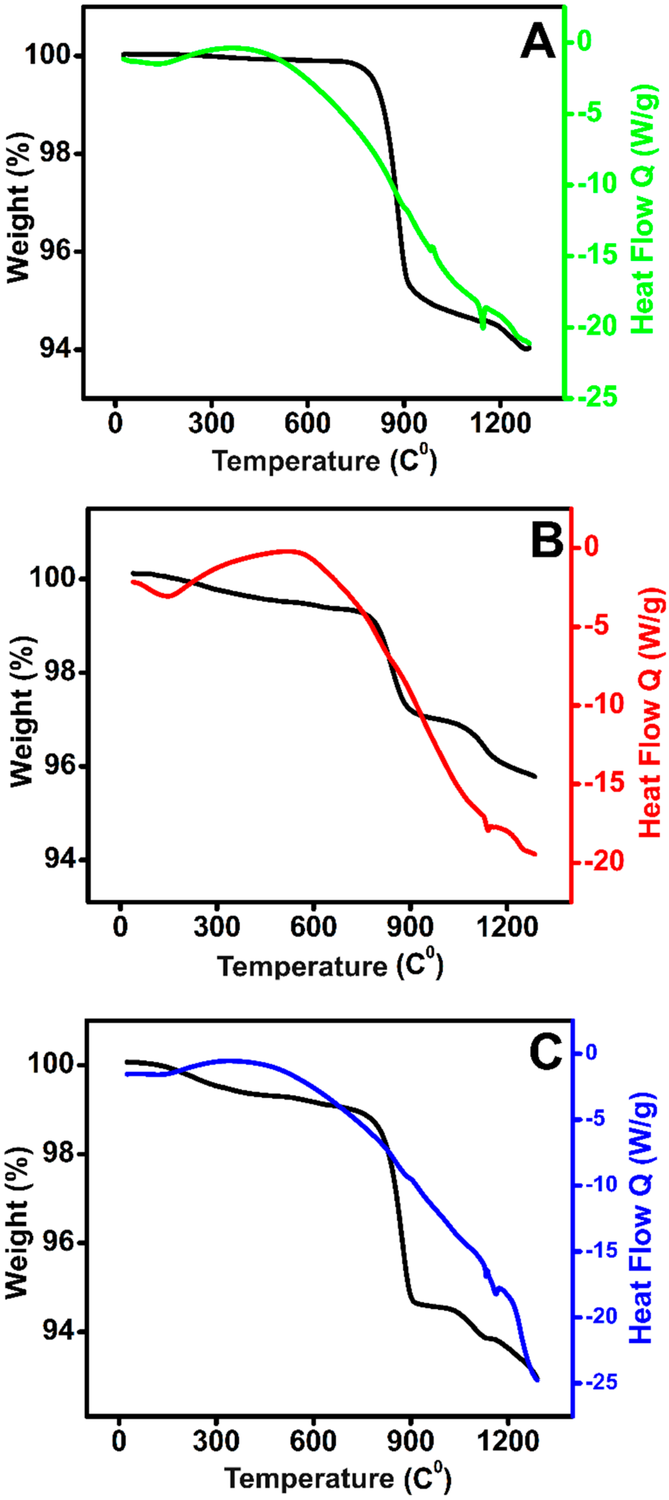

Figure S2. TGA spectra of the LCF NPs (a) + glycerin and eight hour standing time S-013; (b) + glycerin, S-009; and (c) + propylene glycol S-028, respectively.

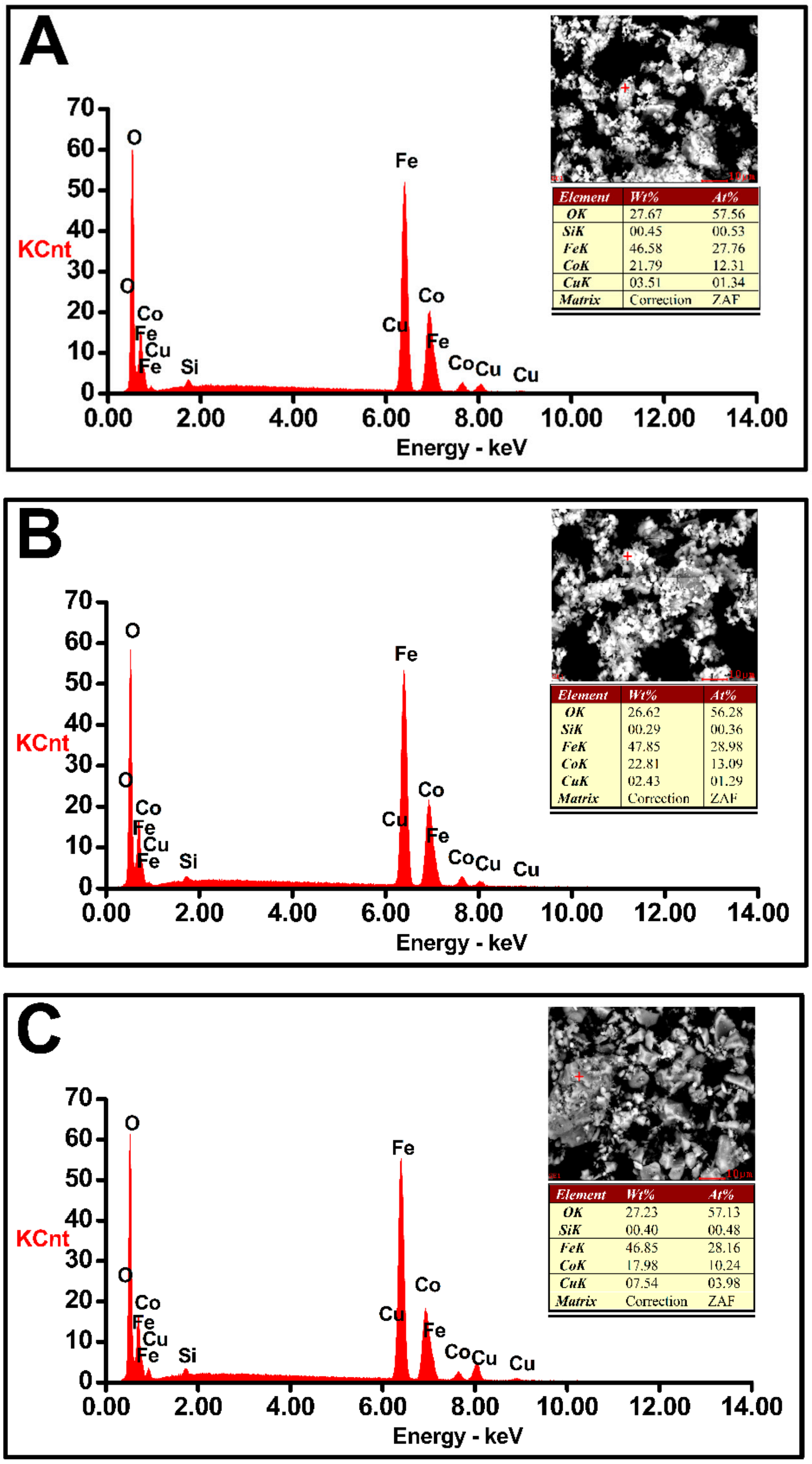

A

Element	Wt%	At%
OK	27.67	57.56
SiK	00.45	00.53
FeK	46.58	27.76
CoK	21.79	12.31
CuK	03.51	01.34
Matrix	Correction	ZAF

B

Element	Wt%	At%
OK	26.62	56.28
SiK	00.29	00.36
FeK	47.85	28.98
CoK	22.81	13.09
CuK	02.43	01.29
Matrix	Correction	ZAF

C

Element	Wt%	At%
OK	27.23	57.13
SiK	00.40	00.48
FeK	46.85	28.16
CoK	17.98	10.24
CuK	07.54	03.98
Matrix	Correction	ZAF

Figure S3. SEM images and quantitative results obtained from EDS analysis of synthesized LCF NPs. A. S-013; B. S-009 and C. S-028 samples respectively

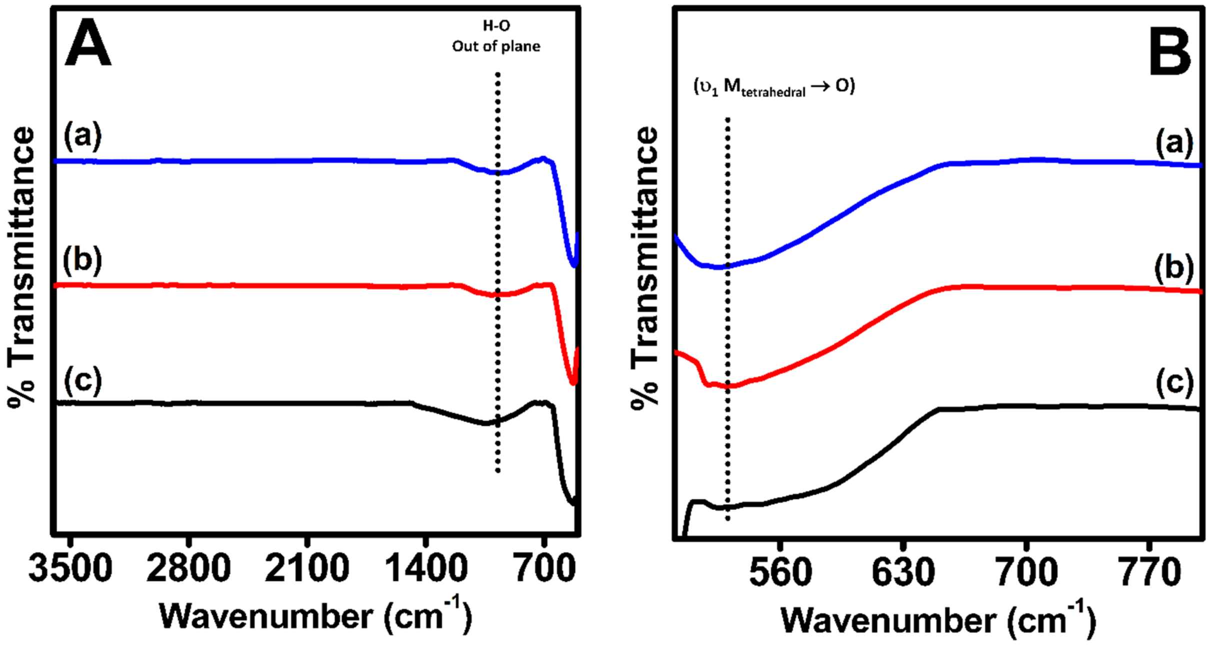

Figure S4. FTIR spectra of LCF NPs in the frequency range of **(A)** 500–3600 cm^{-1}, (B) 500–800 cm^{-1}. Effect of hydroxylated agents and standing time before calcination. (a) + glycerin, S-009; (b) + glycerin and eight hour standing time S-013; and (c) + propylene glycol S-028, respectively.

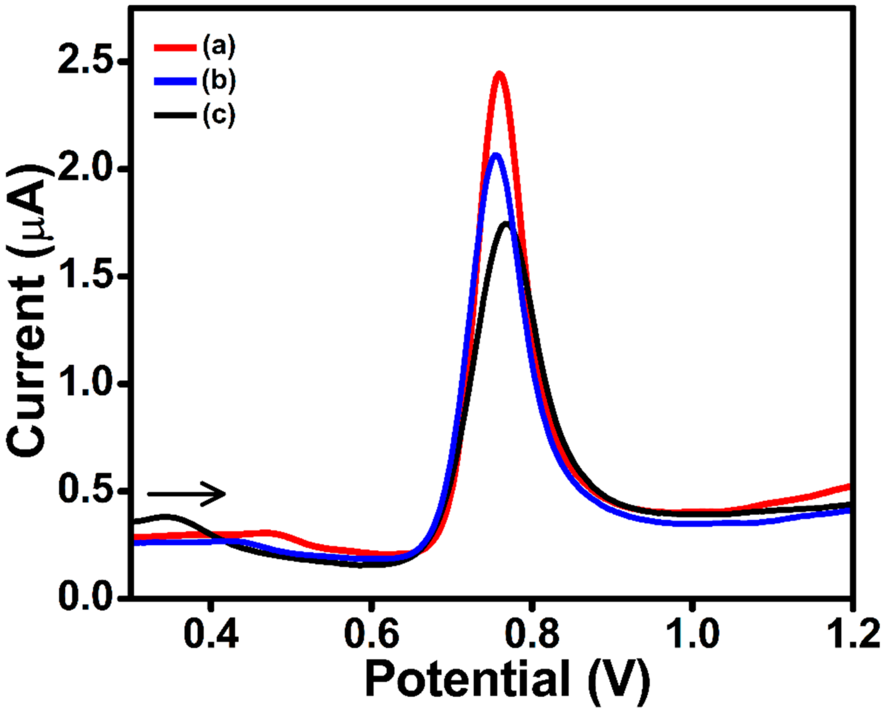

Figure S5. DPVs of 20.0 μM AC in 0.1 M PBS at pH2.5. (a) The S-013/GCE electrode (red), (b) the GCE bare electrode (blue) and (c) the S-028/GCE electrode (black) respectively. The DPV specifications were 0.3 ≤ Epa ≤ 1.2 V at a step potential of 5 mV, pulse amplitudes of 100 mV, pulse times of 50 ms, and a scan rate of 10 mV s^{-1}.

$$I_{\mathrm{p}} = 2.69 \times 10^5\, ACn^{3/2}D^{1/2}v^{1/2} \quad \text{(S1)}$$

where I_p is the anodic or cathodic peak current (A), A is the electroactive area (cm^2), C is the molar concentration of the redox substance, n is thenumber of the transferred electron in the redox reaction, D is the diffusion coefficient of redox probe molecule ($cm^2\,s^{-1}$) and v is the scan rate (V s^{-1}).

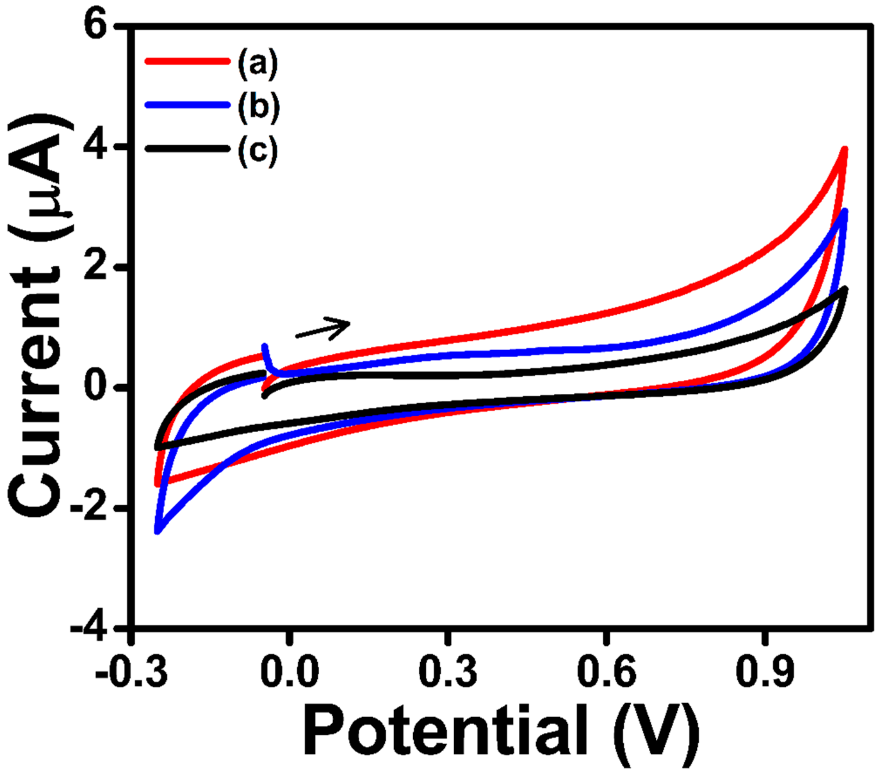

Figure S6. The CVs of the bare electrode, the S-013/GCE, GCE and S-028/GCE electrode in the absence of AC in 0.1M PBS at pH 2.5, *v*: 0.05 Vs^{-1}.

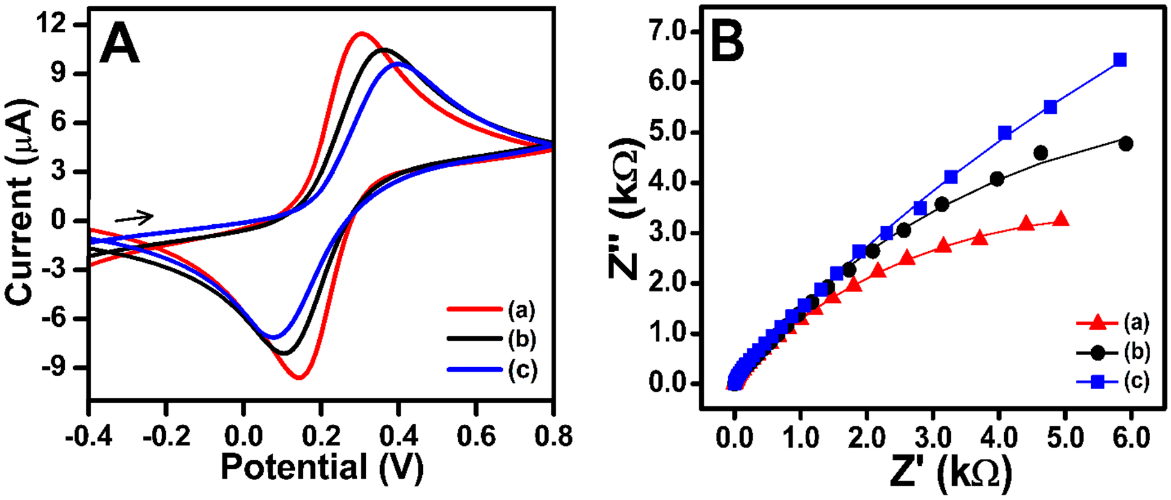

Figure S7. A. Cyclic voltammograms CV at v: 0.1Vs^{-1} and **B.** Electrochemical Impedance Spectroscopy EIS respectively, of 2.5 mM $[Fe(CN)_6]^{3-/4-}$ in 0.1 M KCl, at (a) S-013/GCE, (b) GCE bare electrode, (c) S-028/GCE electrode.

$$K^{\circ} = \frac{RT}{F^2 R_{\mathrm{ct}} A C} \quad \text{(S2)}$$

Where R is the global gas constant (8.314 J K^{-1} mol^{-1}), T is the thermodynamic temperature (298.15 K), F is the Faraday constant (96,485C mol^{-1}), Rct is the electron transfer resistance (Ω), A is the electrode surface area (cm^2), C is the $[Fe(CN)_6]^{3-/4-}$ solution concentration (2.5 mM) and K^0 represents the rate constant of a standard heterogeneous electron transfer (cm s^{-1}).

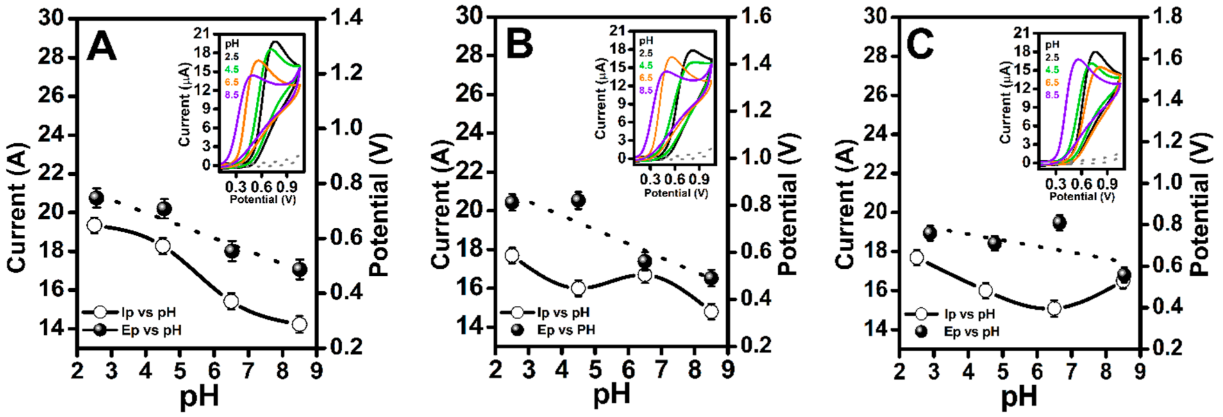

Figure S8. Effect of pH values on the peak potential (dash line) and peak current (solid line) of AC on the LCF NPs/GCE electrodes at various pH values, *v*: 0.05 Vs^{-1}. **A.** S-013/GCE, **B.** GCE bare and **C.** S-028/GCE electrodes. Insert: cyclic voltammograms of solution containing 2.5 mM AC + PBS buffer in the pH range of 2.5–8.5 at the LCF NPs/GCE.

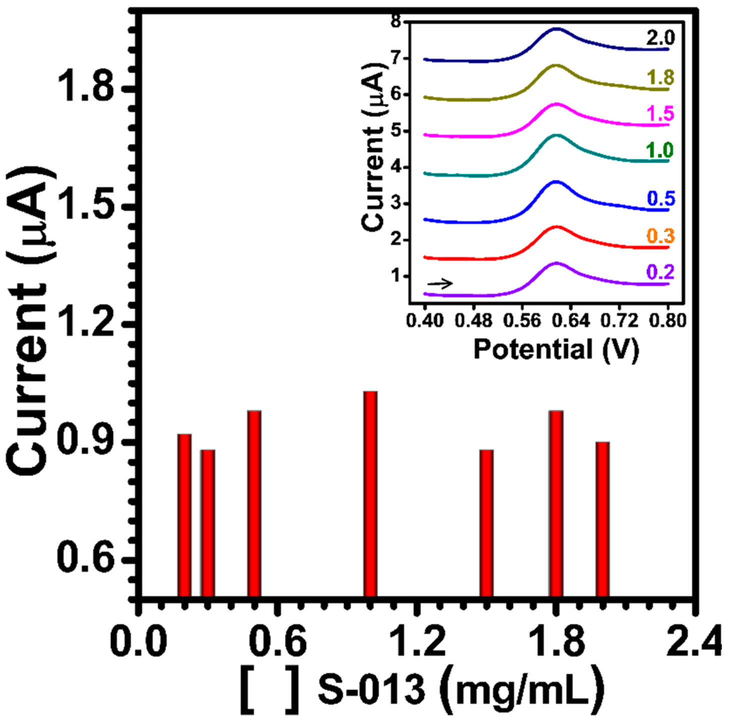

Figure S9. Effect of S-013 nanoparticles concentration on AC response using S-013/GCE electrode with 10.0 μM AC in 0.1 M PBS. Insert show additions of 0.2, 0.3, 0.5, 1.0, 1.5, 1.8 and 2.0 mg mL^{-1} of S-013 nanoparticles, respectively.

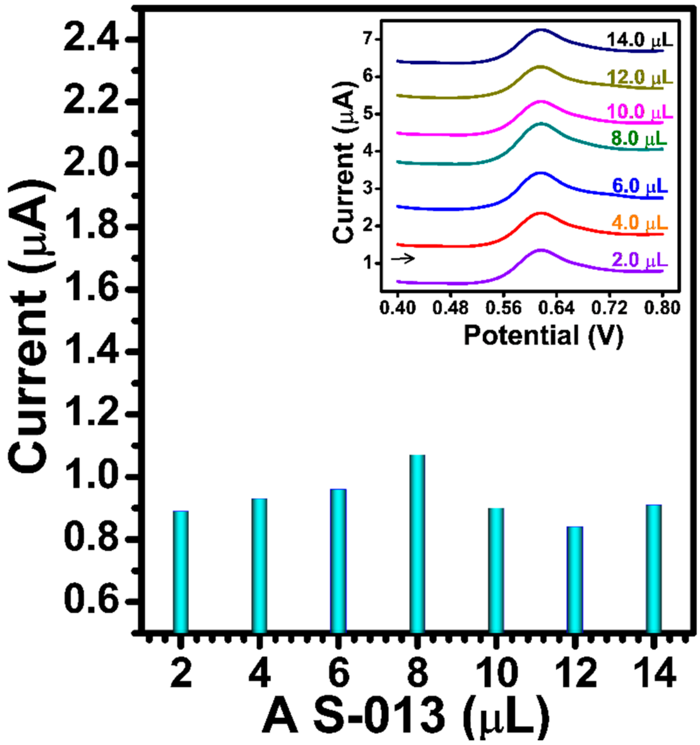

Figure S10. Effect of S-013 amount on AC response using the S-013/GCE electrode, with 10.0 μM AC in 0.1 M PBS. Insert show additions of 2.0, 4.0, 6.0, 8.0, 10.0, 12.0 and 14.0 μL of S-013 nanoparticles, respectively.

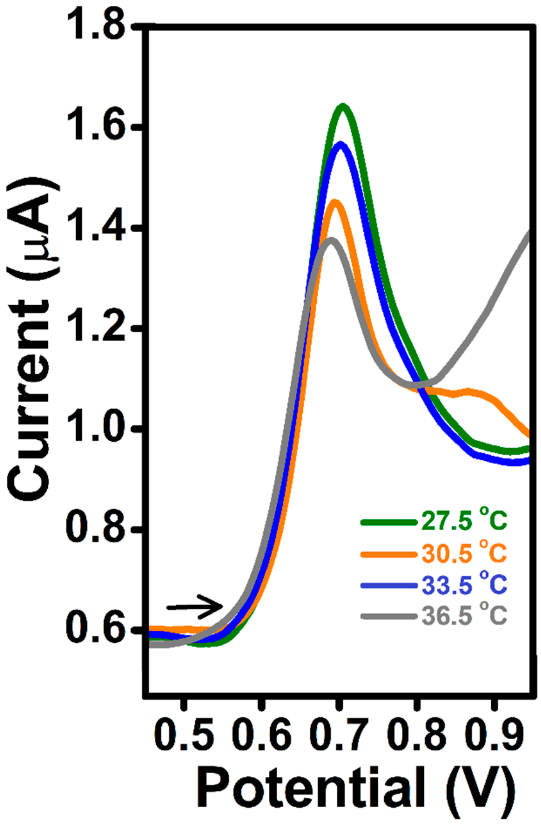

Figure S11. DPV signals from 10 μM AC in 0.1 M PBS. Effect of solution temperature.

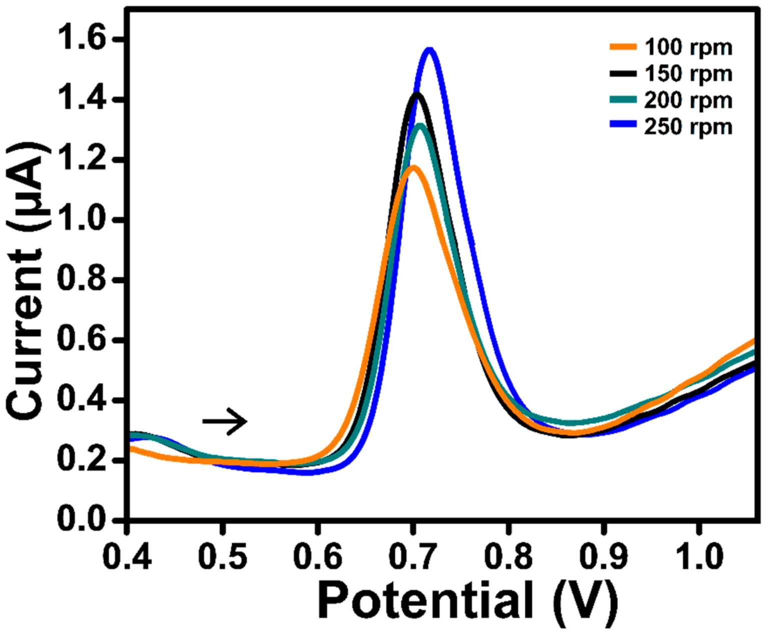

Figure S12. DPV signals from 10 μM AC in 0.1 M PBS. Effect of solution stirring speeds.

Table S1. Comparison of the electrocatalytic performance of different modified electrodes for the determination of AC.

Modified electrode	Technique	Linear range (μM)	Detection limit (μM)	References
Graphene–chitosan/GCE	DPV	1–100	0.3	87
MWNT/GCE	DPV	5–100	2.4	88
TiO2-graphene/poly(methyl red)/GCE	DPV	0.25–50	0.025	89
Nano-TiO2/poly(acid yellow 9)/GCE	DPV	12–120	0.2	90
C60-modified glassy carbon electrode	DPV	50–1500	50.27	91
Poly(taurine)/MWCNT/GCE	DPV	1–100	0.500	92
Graphene/GCE	DPV	0.1–20	0.032	93
$Ba_{1.0}Co_{1.22}Fe_{1.41}O_{18.11}$/GCE	DPV	1-12	0.530	94
rGO-PEDOT NT/GCE	DPV	1–35	0.400	95
Fe3O4@Au–S–Fc/GS-chitosan/GCE	DPV	0.4–32	0.050	96
4-ABA/ERGO/GCE	DPV	0.1–65	0.010	97
$Co_{0.87}Cu_{0.13}Fe_2O_4$/GCE	DPV	10-100	0.099	**This work**

Reference

87. Zheng, M.X.; Gao, F.; Wang, Q.X.; Cai, X.L.; Jiang, S.L.; Huang, L.Z.; Gao, F. Electrocatalytic oxidation and sensitive determination of acetaminophen on glassy carbon electrode modified with graphene–chitosan composite *Mater. Sci. Eng. C*, **2013**, *33*, 1514-1520
88. Wan, Q.J.; Wang, X.W.; Yu, F.; Wang, X.X.; Yang, N.J. Effects of capacitance and resistance of MWNT-film coated electrodes on voltammetric detection of acetaminophen *J. Appl. Electrochem.*, **2009**, *39*, 1145-1151
89. Xu, C.; Huang, K.; Fan, Y.; Wu, Z.; Li J. Electrochemical determination of acetaminophen based on TiO_2-graphene/poly(methyl red) composite film modified electrode *J. Mol. Liq.*, **2012**, *165*, 32-37
90. Kumar, S.A.; Tang, C.F.; Chen S.M. Electroanalytical determination of acetaminophen using nano-TiO_2/polymer coated electrode in the presence of dopamine *Talanta*, **2008**, *76*, 997-1005
91. Goyal, R.N.; Singh, S.P. Voltammetric determination of paracetamol at C60-modified glassy carbon electrode. *Electrochim. Acta* **2006**, *51*, 3008–3012.
92. Wan, Q.; Wang, X.; Yu, F.; Wang, X.; Yang N. Poly(taurine)/MWNT-modified glassy carbon electrodes for the detection of acetaminophen. *J. Appl. Electrochem.*, **2009**, *39*, 785-790
93. Kang, X.; Wang, J.; Wu, H.; Liu, J.; Aksay, I.A.; Lin Y. A graphene-based electrochemical sensor for sensitive detection of paracetamol. *Talanta*, **2010**, *81*, 754-759
94. Granja-Banguera C.P.; Silgado-Cortázar D.G.; Morales-Morales J.A. Transition Metal Substituted Barium Hexaferrite Modified Electrode: Application as Electrochemical Sensor of Acetaminophen. *Molecules* **2022**, *27*, 1550
95. Huang, T.Y.; Kung, C.W.; Wei, H.Y.; Boopathi, K.M.; Chu, C.W.; Ho, K.C. A high performance electrochemical sensor for acetaminophen based on a rGO-PEDOT nanotube composite modified electrode. *J. Mater. Chem. A*, **2014**, *2*, 7229-7237
96. Liu, M.; Chen, Q.; Lai, C.L.; Zhang, Y.Y.; Deng, J.H.; Li, H.T.; Yao S.Z. A double signal amplification platform for ultrasensitive and simultaneous detection of ascorbic acid, dopamine, uric acid and acetaminophen based on a nanocomposite of ferrocene thiolate stabilized Fe3O4@Au nanoparticles with graphene sheet *Biosens. Bioelectron.*, **2013**, *48*, 75-81
97. Zhu, W.; Huang, H.; Gao, X.; Ma, H. Electrochemical behavior and voltammetric determination of acetaminophen based on glassy carbon electrodes modified with poly(4-aminobenzoic acid)/electrochemically reduced graphene oxide composite films. *Materials Science and Engineering: C*, **2014**, *45*, 21-28

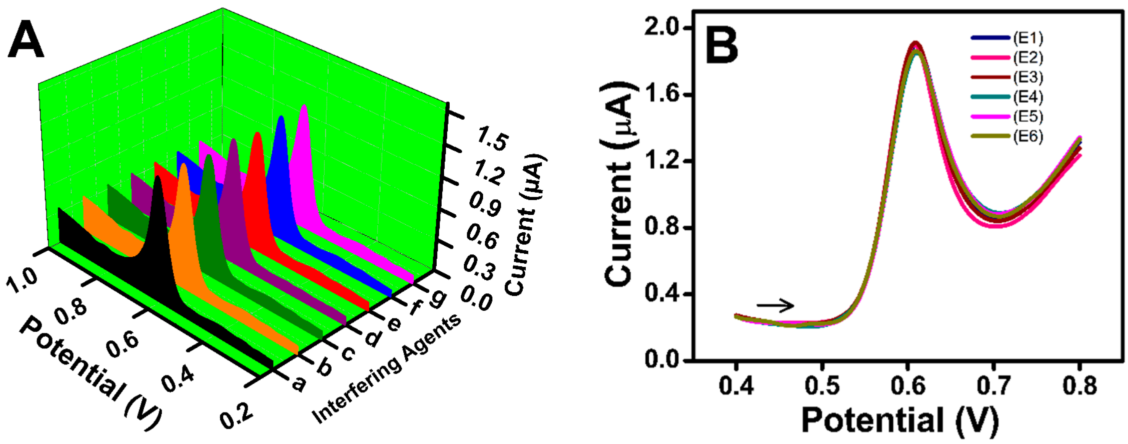

Figure S13. A. DPVs of 5 μM AC with 100-fold concentrations of (a) K^+, (b) Ca^{2+}, (c) Na^+, (d) Mg^{2+}, (e) ascorbic acid, (f) uric acid and (g) glucose. **B**. The reproducibility curves of 10 μM AC at six different S-013/GCE electrodes, E1 to E6 respectively

Table S2. The effect of different interferences at S-013/GCE in the determination of AC.

	Interference	Concentration (mol L^{-1})	Peak change (%)
a	K^+	$5x10^{-4}$	2.17
b	Ca^{2+}	$5x10^{-4}$	2.04
c	Na^+	$5x10^{-4}$	2.21
d	Mg^{2+}	$5x10^{-4}$	2.26
e	Ascorbic Acid	$5x10^{-4}$	2.38
f	Uric Acid	$5x10^{-4}$	2.46
g	Glucose	$5x10^{-4}$	2.14
